# Midkine promotes renal fibrosis by stabilizing C/EBPβ to facilitate endothelial-mesenchymal transition

**DOI:** 10.1038/s42003-024-06154-0

**Published:** 2024-05-07

**Authors:** Cuidi Xu, Juntao Chen, Lifei Liang, Siyue Chen, Xinhao Niu, Ruirui Sang, Cheng Yang, Ruiming Rong

**Affiliations:** 1 Department of Urology, Zhongshan Hospital, Fudan University; Shanghai Key Laboratory of Organ Transplantation, Shanghai, 200032 China; 2 Department of Transfusion, Zhongshan Hospital, Fudan University, Shanghai, 200032 China; 3 Zhangjiang Institute of Fudan University, Shanghai, 201203 China

**Keywords:** Renal fibrosis, Ubiquitylation

## Abstract

Numerous myofibroblasts are arisen from endothelial cells (ECs) through endothelial to mesenchymal transition (EndMT) triggered by TGF-β. However, the mechanism of ECs transforms to a different subtype, or whether there exists an intermediate state of ECs remains unclear. In present study, we demonstrate Midkine (MDK) mainly expressed by CD31 + ACTA2+ECs going through partial EndMT contribute greatly to myofibroblasts by spatial and single-cell transcriptomics. MDK is induced in TGF-β treated ECs, which upregulates C/EBPβ and increases EndMT genes, and these effects could be reversed by siMDK. Mechanistically, MDK promotes the binding ability of C/EBPβ with ACTA2 promoter by stabilizing the C/EBPβ protein. In vivo, knockout of Mdk or conditional knockout of Mdk in ECs reduces EndMT markers and significantly reverses fibrogenesis. In conclusion, our study provides a mechanistic link between the induction of EndMT by TGF-β and MDK, which suggests that blocking MDK provides potential therapeutic strategies for renal fibrosis.

## Abstract (web summary)

This study focuses on how endothelial cells contribute to ECM accumulation of renal fibrosis and suggests that MDK promotes ACTA2 expression by stabilizing the C/EBPβ protein.

## Introduction

Interstitial fibrosis characterized by excessive formation of extracellular matrix (ECM) produced by myofibroblasts is considered a common result of multiple end-stage kidney diseases^1,2^. Diverse cellular origins of myofibroblasts have reported, including epithelial cells (EPI), endothelial cells (ECs), bone marrow-derived fibrocytes, and macrophage^3–6^. The contribution of ECs as a critical origin of myofibroblasts varies greatly in different organs. Zhang et al. found that ECs did not contribute to myofibroblasts in cardiac fibrosis^7^. In contrast, in situ evidence of endothelial to mesenchymal transition (EndMT) was obtained in lung fibrosis result from human pulmonary arterial hypertension^8^. By using endothelial lineage tracing, Zeisberg et al. first found that up to 30–50% of activated fibroblasts may arise from ECs via EndMT during renal fibrosis^9^. Although a part of studies implicated that activated myofibroblasts may be derived from pericytes adjacent to ECs^10^. LeBleu et al. suggested pericytes do not contribute to myofibroblasts in kidney fibrosis^11^. Therefore, the role of ECs as a target in kidney fibrotic conditions deserves more investigation.

There are two phenomena of EndMT, partial EndMT and complete EndMT. Complete EndMT is a total change of cell identity which involves complete shedding of endothelial trait accompanied with acquisition of mesenchymal trait. ECs going through partial EndMT appear to be an intermediate state which is a reversible period along the endothelial-mesenchymal spectrum^12–14^. The mesenchymal (myofibroblast activity) and endothelial phenotype of ECs depends on substance being stimulated. Transforming growth factor β (TGF-β) is an important mediator of EndMT in fibrosis. However, the underlying mechanism is still unclear.

Midkine (MDK) plays a crucial role in fibrogenesis, but its source in vivo has not been established^15,16^. Strong MDK expression was observed in patients with diabetic nephropathy^17^ and other inflammatory conditions^18,19^. Liu et al. reported that promoted expression of MDK accelerated the pathological damage and fibrosis in diabetic kidney diseases by increasing neutrophil extracellular trap^20^. Similarly, Misa et al. found knockout of *Mdk* significantly ameliorated lung fibrosis with lower expression of both collagen and α-Smooth Muscle Actin (ACTA2)^21^. Extracellular MDK can be transported into the cytosol by endocytosis, and undergoes proteasomal degradation in the nucleus^22^. Extracellular MDK is known to bind transmembrane receptors in an autocrine and paracrine manner and activate intracellular signaling. However, the function of intracellular MDK remains unclear.

In our study, we investigate a new mechanism of MDK in mediating EndMT. MDK mainly expressed by CD31^+^ACTA2^+^ ECs during partial EndMT contributes greatly to myofibroblasts and promotes renal fibrosis through stabilizing C/EBPβ, which subsequently activate ACTA2 expression by directly targeting its promoter region. Decreasing the cellular MDK by siMDK prevents the activation of ACTA2 in ECs. Our work suggests that blocking MDK provides a potential therapeutic strategy for renal fibrosis.

## Results

### ECs contribute greatly to myofibroblasts through EndMT

**Fig. 1:**
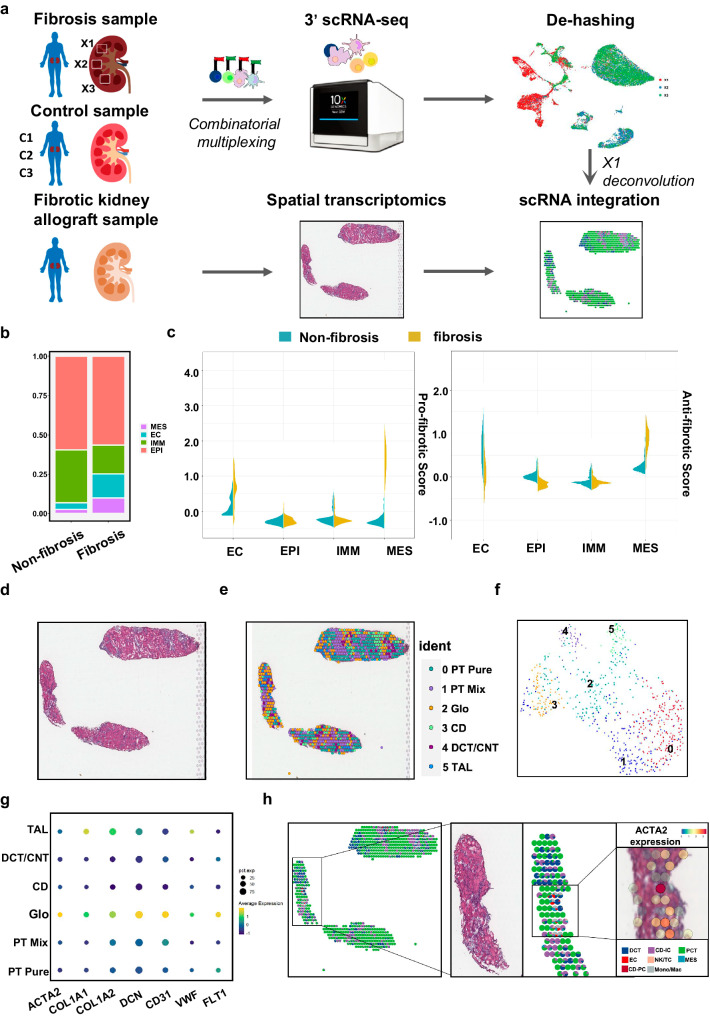
ECs contribute to myofibroblasts through EndMT in renal fibrosis. **a** Overview of spatial transcriptomics and single cell design. **b** Cell proportion in non-fibrosis and fibrosis patients. **c** ECM-related genes score of EPI, IMM, EC, and MES. **d** H&E staining of the patient reference nephrectomy. **e**, **f** A version unsupervised clusters overlaid on the nephrectomy. **g** Dot plot for the expression levels of EC markers and ECM markers. **h** Deconvolution of X1 scRNA data to spatial map. Each pie chart represents the contribution of cell types from X1 scRNA data set to the spatial transcriptomic signature of each spot. Only cell types in X1 contributing to at least 10% of spot are displayed.

Single-cell transcriptomic technologies are enriching our understanding of cellular heterogeneity and providing novel insights into fundamental renal biology such as the cell differentiation and the cell communication. We collected six kidney tissues from four patients, three fibrosis tissues (X1, X2, X3), and three non-fibrosis tissues from three patients (C1, C2, C3) for single-cell sequencing (Fig. 1a). EPI, immune cells (IMM), ECs and mesenchymal cells (MES) were identified from these two groups based on respective markers(Fig. 1b, S1a, b). Myofibroblasts are major players in the excessive deposition of ECM. To further explore which cell type contributes to ECM accumulation in kidney fibrosis, we established a single cell ECM-related expression score including collagens, collagen-degrading enzymes (Matrix Metalloproteinases family, MMP family) and ECM turnover factors (Tissue Inhibitors of Metalloproteinases, TIMP family). We have separated these genes into pro-fibrotic group (collagens, TIMPs) and anti-fibrotic group (MMPs). We demonstrated that ECs exhibited higher score of pro-fibrotic genes in fibrosis group and exhibited slightly lower score of anti-fibrotic genes in fibrosis group (Fig. 1c).

Single-cell transcriptomes lose their spatial positional information during tissue dissociation. Therefore, we collected tissue from a 53-years-old woman with interstitial fibrosis for spatial transcriptomics. To spatially resolve cell populations, the spatial transcriptomics data was integrated with the scRNA-seq data of X1 tissue (Fig. 1a). After DNA synthesis and ST library construction, we detected 676 spots overlaid upon the histological image of the kidney section at a median depth of 12,940 UMIs, including 4086 genes per spot. These spots were clustered in an unsupervised manner according to gene expression and identified according to known kidney regions (Figure S1c–i). Referring to the known marker genes of glomeruli and tubular system (Fig. S1c–i), six clusters (proximal tube, PT, glomeruli, Glo, collecting ducts, CD, distal convoluted tubules, DCT, connecting tubules, CNT, thick ascending limb, TAL) were generated by Space Ranger (Fig. 1d–f). Surprisingly, the genes distribution in H&E section of MES and ECM markers were almost the same as EC markers (Fig. 1g). We used the prediction scores to transfer annotation from single-cell to spatial transcriptomes. ECs were found to co-located with MES, which is widely known as an origin of myofibroblasts (ACTA2^+^) (Fig. 1h). Therefore, we hypothesized that ECs may contribute to ECM accumulation by EndMT.

### The Mdk expressed by ECs involves in EndMT of renal fibrosis

**Fig. 2:**
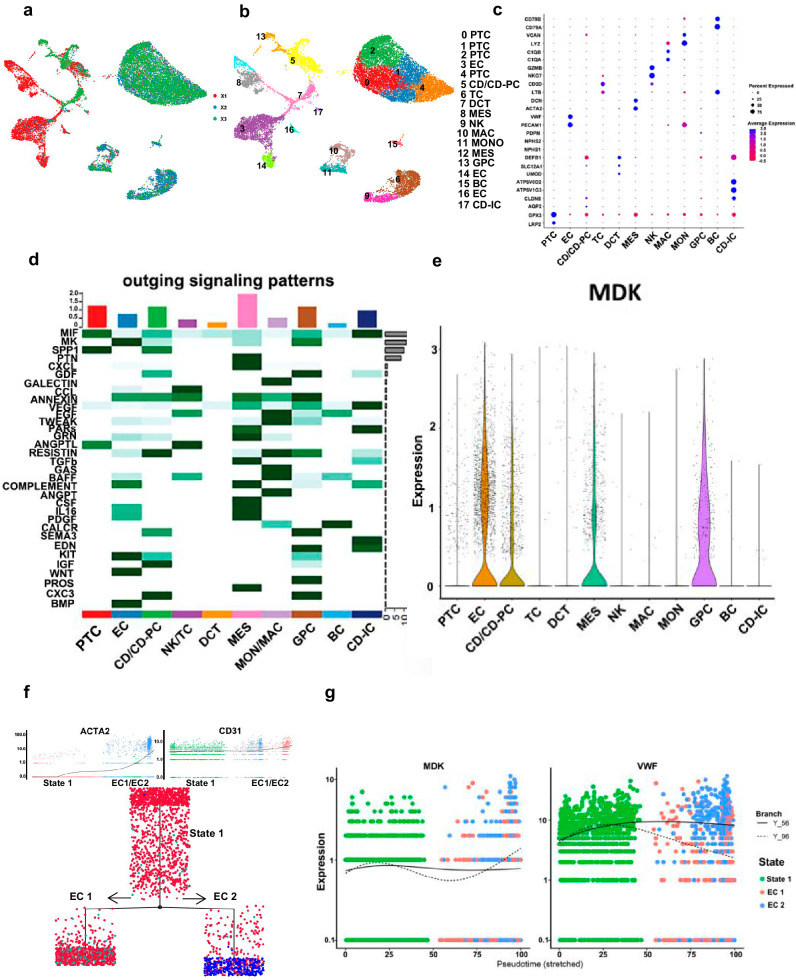
The Mdk expressed by ECs involves in EndMT of renal fibrosis. **a**, **b** Unsupervised clustering of fibrosis patient (X1, X2, X3) demonstrates 18 clusters shown in the umap map. **c** Expression of cell markers used to classify clusters. **d** Heatmap shows the key ligands outgoing signaling pattern of different cells as secreting cells. The darker the color, the greater the role signaling play. **e** Violin plots for the expression of the MDK gene in all cells. **f** Pseudotime analysis showed EC differentiated into two states during fibrogenesis. **g** Branch analysis showed the emergence of increasing level of MDK represent the initial transformation from EC1 to EC2.

Based on the known cell markers, 18 clusters of cells were identified in 3 fibrotic samples, including epithelial cells (proximal tubules cells, PTC, collecting duct principal cells, CD-PC, collecting duct intercalated cells, CD-IC, distal convoluted tubules cells, DCT, glomerular parietal epithelial cells, GPC), IMMs (B lymphocytes, BC, T lymphocytes, TC, natural killer cells, NK, monocytes, MON, macrophage, MAC), EC and MES (Fig. 2a–c). CellChat analysis was subsequently performed to understand how signals were activated during fibrogenesis. The signal network indicated that the MK (also known as MDK) signal was the most active pathway produced by ECs (Fig. 2d, Fig. S2a). After evaluation the transcription level of *Mdk* gene at a single-cell level, we found ECs had the highest expression. Other cells including MES had low or no expression of *Mdk* gene in fibrosis samples (Fig. 2e). Therefore, ECs possibly produced the greatest level of MDK in renal fibrosis.

To investigate whether ECs have a tendency to transform into myofibroblasts during fibrosis, we simulated partial EndMT by using Monocle. We separated 3 clusters of ECs from X1, X2, X3 for pseudotime analysis and performed DoubletFinder to rule out doublets in EC clusters (Fig. S2b). The pseudotime analysis illustrated that ECs differentiated into two states: EC1 and EC2 (Fig. 2f). EC1 which exhibited endothelial phenotype (CD31^+^ACTA2^−^) contributed greatly in original state (state 1). EC2 displayed a mesenchymal phenotype (CD31^+^ACTA2^+^). The expression of endothelial markers fms related tyrosine kinase 1 (FLT1) and von Willebrand factor (VWF) decreased with the transition from EC1 to EC2 (Fig. S2c). Contrarily, Decorin (DCN), TIMP2, and TIMP3 expressions increased with the transition from EC1 to EC2 (Fig. 2f, S2d). The branch analysis showed an increasing level of MDK from EC1 to EC2 (Fig. 2g). Therefore, these results indicated that CD31^+^ACTA2^+^ ECs can be seen as an intermediate state from partial EndMT to complete EndMT, and MDK may play a crucial role in partial EndMT.

### CD31^+^ACTA2^+^ECs increasingly expresses MDK during EndMT

**Fig. 3:**
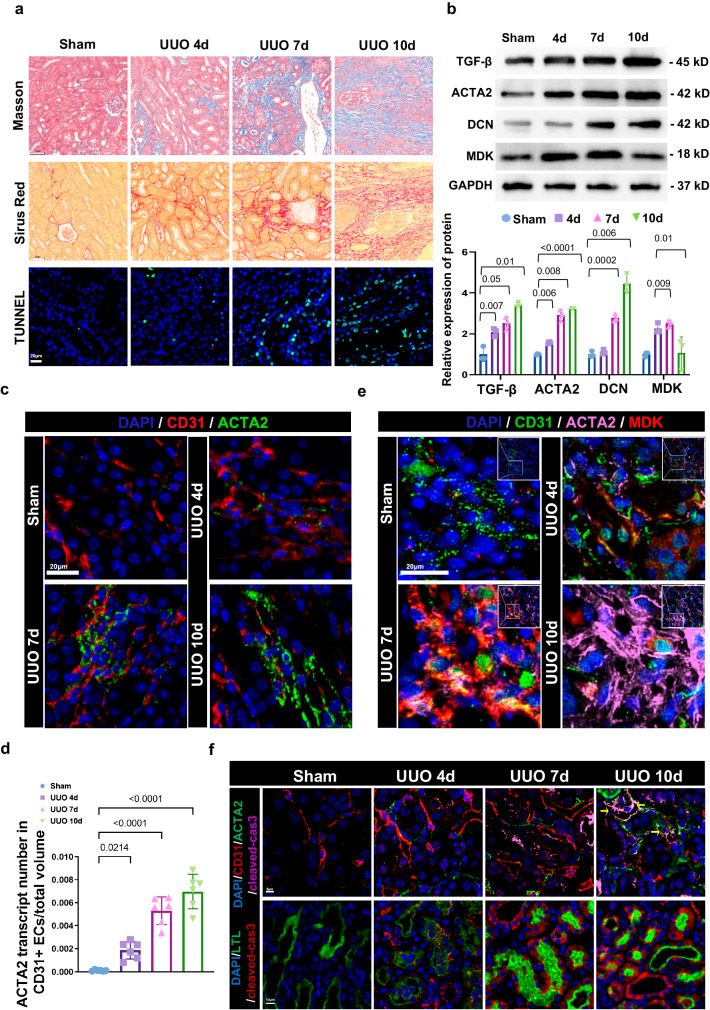
CD31+ACTA2+ECs increasingly expresses MDK during EndMT. **a** Masson staining and Sirius red staining in UUO 0d, 4d, 7d, 10d mice. *n* = 6 for all groups. Original magnification, ×20. Scale bars, 50 μm. **b** WB analysis of pro-fibrotic markers and MDK in UUO 0d, 4d, 7d, 10d mice. *n* = 3 for all groups. Each data point represents one animal. Three biological repeated immunoblots have been performed. **c**, **d** Representative image of CD31 and ACTA2 IF staining shows CD31 + ACTA2+ ECs elevation in UUO 4d, 7d, 10d. *n* = 6 for all groups. Original magnification, ×40. Scale bar, 20 μm. Each data point represents one animal. **e** Representative image of CD31, ACTA2, and MDK IF staining shows CD31^+^ACTA2^+^ ECs secret MDK. *n* = 6 for all groups. Original magnification, ×40. Scale, 20 μm. **f** Representative image of cleaved caspase3 with CD31, ACTA2 or LTL shows apoptosis of CD31^+^ACTA2^+^ ECs and tubular cells in UUO 4d, 7d, 10d. *n* = 6 for all groups. Original magnification, ×40. Scale, 20 μm. *P* values were calculated using an unpaired Student’s *t* test for 2 groups or ANOVA with Tukey’s test for multiple comparison. All the error bars above denote standard deviation.

We performed unilateral uretera obstruction (UUO) in mice to replicate fibrogenesis in vivo. We evaluated the content of collagen in kidney section as an indicator for renal fibrosis by using Masson’s trichrome staining and Sirius red staining, and cell apoptosis using TUNEL staining. Significant increases of fibrotic tissue and cell apoptosis in UUO mice were observed compared to sham mice (Fig. 3a). WB experiment also showed the profibrotic markers TGF-β, ACTA2, and DCN were increased in UUO group. However, the expression of MDK in UUO 10d was lower compared with that in UUO 7d (Fig. 3b), which may result from the complete EndMT in UUO 10d. To further demonstrate the MDK expression in ECs during fibrosis, IF staining was performed. In the sham group, we found CD31^+^ ECs did not co-express ACTA2 expression, which revealed that few numbers of CD31^+^ACTA2^+^ ECs were observed in normal kidney. However, ~19.63% of CD31^+^ ECs expressed ACTA2 in 4d UUO group and up to 51.61% of CD31^+^ ECs expressed ACTA2 in 7d UUO group (Fig. 3c, d). To further examine ECs express MDK, we performed ECs markers and MDK staining in protein level. IF staining of CD31, ACTA2, and MDK demonstrated that MDK was expressed by CD31^+^ACTA2^+^ ECs and the expression of MDK from CD31^+^ACTA2^+^ ECs was significantly increased in 4d group and reached a peak level in the 7d group (Fig. 3e). In 10d UUO group, kidney exhibited fibrotic phenotype with highest expression of ACTA2, but the number of CD31^+^ ECs and expression of MDK obviously reduced compared to the 7d UUO group (Fig. 3e). Since CD31 is also expressed in platelets and leukocytes, we also performed IF staining of CD31, VWF, ACTA2 and MDK in 4d and 7d, cells co-expressing CD31 and VWF were seen as ECs, the co-location of CD31, VWF, ACTA2 and MDK further confirmed MDK is expressed by ECs (Fig. S3a).

Cell apoptosis is closely associated with EndMT. MDK is also known as an anti-apoptotic factor^23^. We performed IF staining of CD31, ACTA2, and cleaved caspase-3 to explore the apoptosis of CD31^+^ACTA2^+^ ECs. We found the co-expression of CD31, ACTA2, and cleaved caspase-3 is hardly seen in 0d, 4d, and 7d except 10d UUO treatment group (Fig. 3f). However, the co-expression of Lotus tetragonolobus lectin (LTL) and cleaved caspase-3 were increased with fibrogenesis in UUO mice (Fig. 3f). It seems that cellular apoptosis during the process of fibrosis is manifested with the apoptosis of tubular cells rather than ECs. Conclusively, we proved that MDK increasingly expressed by CD31^+^ACTA2^+^ ECs during EndMT may be involved in the development of renal fibrosis.

### TGF-β induces MDK expression in ECs via SMAD2/3 signaling

**Fig. 4:**
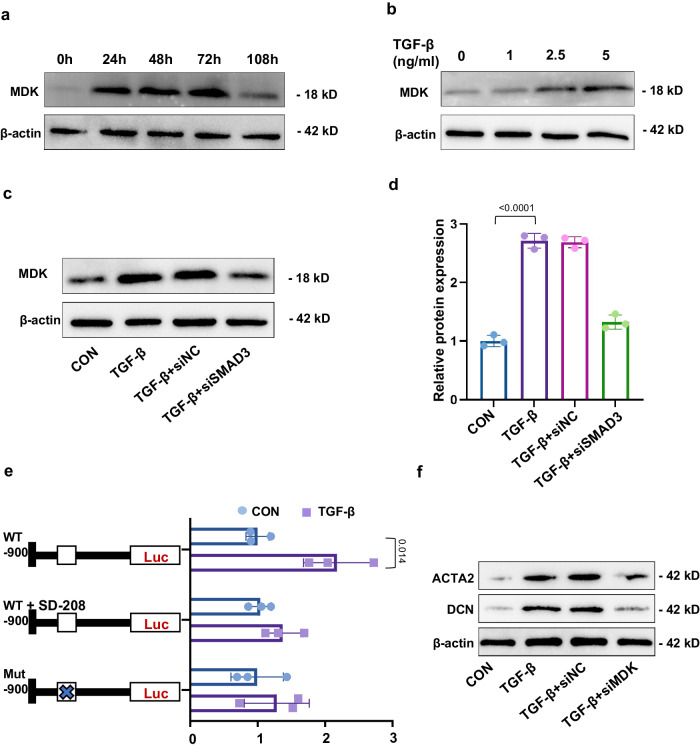
TGF-β induces MDK expression in ECs via SMAD3 signaling. **a** WB analysis of MDK in HUVECs treated with TGF-β for 0–108 h. **b** MDK levels in HUVECs treated with or without TGF-β. **c**, **d** WB experiment shows enhanced level of MDK can be reserved by SMAD3 knockdown. *n* = 3 biologically independent samples/experiments. **e** Left: Linear map schematic of the putative SBE at promoter of the *Mdk* gene, with the mutated luciferase (Luc) construct. Right: Graph shows the luciferase assay results. *n* = 3 biologically independent experiments. Unpaired two-tailed Student’ *t* test. **f** WB experiment shows the upregulation levels of ACTA2 and DCN induced by TGF-β can be reserved by MDK knockdown. *P* values were calculated using an unpaired Student’s *t* test for 2 groups or ANOVA with Tukey’s test for multiple comparison. All the error bars above denote standard deviation.

TGF-β-induced EndMT is involved in organ fibrosis, whereby ECs adopt a mesenchymal phenotype with expressing MES markers. To study whether MDK is stimulated by TGF-β, we examined the protein levels of MDK after TGF-β treatment over time. In HUVECs, TGF-β treatment increased MDK in a time- and concentration-dependent manner (Fig. 4a, b). SMAD proteins are known as signal transducers of TGF-β controlling gene expression. The upregulation of MDK can be reversed by knockdown of SMAD3 (Fig. 4c, d). Bioinformatic analysis displayed a SMAD3-binding element (SBE) (−914 to −953) in the *Mdk* promoter region. Next, we constructed a plasmid to monitor the TGF-β driven transactivaton of a *Mdk*-specific reporter. We found *Mdk* transcription was induced when the promoter constructs contained SBE, but this effect was reversed after treating with SD-208 (a TGF-β/SMAD3 signaling inhibitor). Moreover, *Mdk* transcription could also be reduced when SBE was mutated (Fig. 4e). The efficiency of SMAD3 and MDK siRNA were confirmed in HUVECs (Fig. S3b, c). Then, we downregulated MDK in HUVECs cultured with TGF-β stimulation for 48 h. MDK downregulation result in decreases of the mesenchymal marker ACTA2 and ECM marker DCN (Fig. 4f). Therefore, we concluded that TGF-β/SMAD2/3 signaling mediates MDK expression, which may involve in EndMT process.

### MDK stabilizes C/EBPβ by disrupting C/EBPβ-MDM2 complex

**Fig. 5:**
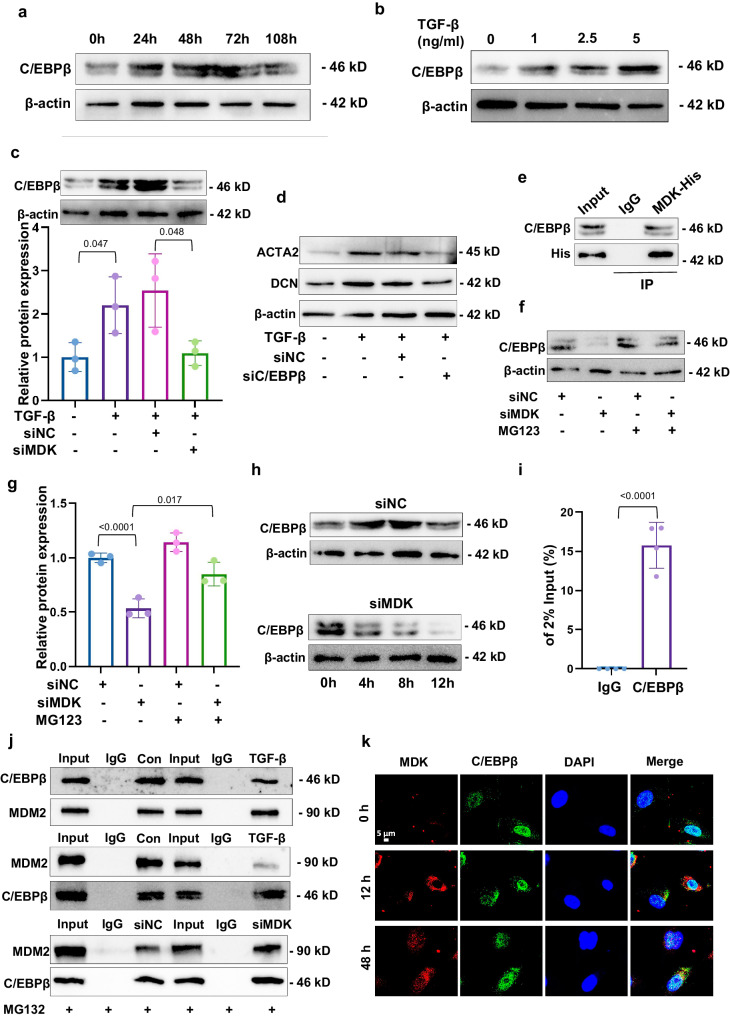
MDK stabilizes C/EBPβ by disrupting C/EBPβ-MDM2 complex. **a** WB analysis of C/EBPβ in HUVECs treated with TGF-β for 0–108 h. **b** C/EBPβ levels in HUVECs treated with or without TGF-β. **c** WB experiment shows the upregulation level of C/EBPβ induced by TGF-β can be reserved by MDK knockdown. Unpaired two-tailed Student’ *t* test. **d** WB experiment shows the upregulation levels of ACTA2 and DCN induced by TGF-β can be reserved by C/EBPβ knockdown. **e** Co-IP experiment shows MDK interacts with C/EBPβ protein. **f**, **g** WB analysis of the effect of MDK knockdown on C/EBPβ with or without MG132 treatment (10 μM, 12 h). Unpaired two-tailed Student’ *t* test. **h** WB analysis of C/EBPβ in MDK knockdown HUVECs after treatment with CHX (10 μg/ml) for indicated times. **i** ChIP-qPCR shows C/EBPβ interacts with the promoter of ACTA2. Values correspond to the ratio between the anti-C/EBPβ antibody immunoprecipitated DNA relative to the IgG immunoprecipitated DNA, *n* = 4 biologically independent samples/experiments. Data are presented as means ± SD, Unpaired two-tailed Student’s *t* test. **j** Co-IP analysis of interaction between C/EBPβ and MDM2 in TGF-β-treated HUVECs transfected with or with siMDK. Three biological repeated immunoblots have been performed. **k** IF staining shows the co-location of MDK and C/EBPβ. All the error bars above denote standard deviation.

Given MDK is not a transcriptional factor which lacks of the ability to directly activate ACTA2, we predicted the possible transcription factor of ACTA2 by online database PROMO, and selected C/EBPβ as a possible target of MDK. We discovered a conserved putative C/EBPβ-binding motif (TTGGGCAA) in promoter region of ACTA2 gene. Therefore, we hypothesized that C/EBPβ contributed to the emergence of CD31^+^ACTA2^+^ ECs by directly binding to ACTA2 promoter. WB analysis showed that the expression of C/EBPβ increased with dose and time of TGF-β treatment, which is same as MDK level in TGF-β-induced EndMT (Fig. 5a, b). To assess the relationship between C/EBPβ activation and MDK in EndMT, we transfected siMDK for loss-of-function experiments. Knockdown of MDK prevented the upregulation of C/EBPβ in response to TGF-β (Fig. 5c). Furthermore, transfection of siC/EBPβ counteracted the effect of TGF-β treatment on the expression of ACTA2 and DCN (Fig. 5d). Then, we further used Co-IP analysis and revealed the interaction between MDK and C/EBPβ (Fig. 5e). siMDK decreased C/EBPβ protein, but not mRNA (Fig. S3d), which suggested that MDK regulates C/EBPβ protein in translational or posttranslational level. ECs were treated with proteasome inhibitor MG132. Silencing of MDK significantly decreased the level of C/EBPβ, whereas MG132 treatment with inhibited MDK displayed minimal changes in C/EBPβ level (Fig. 5f, g). Moreover, we further blocked protein synthesis by using cycloheximide (CHX) and found MDK knockdown disrupted C/EBPβ protein stability (Fig. 5h, S3e). IP results demonstrated that knockdown of MDK significantly increased the ubiquitylation of C/EBPβ in ECs (Fig. S3f). To further test whether C/EBPβ interact with Acta2 promoter region, ChIP-qPCR experiment was applied. We observed the interaction between C/EBPβ and ACTA2 promoter (Fig. 5i). Furthermore, ChIP-qPCR were further perform after knockdown of MDK, result showed that siMDK prevented the interaction between C/EBPβ and Acta2 promoter (Fig. S3g), suggesting that MDK was involved in TGF-β induced EndMT. Therefore, our results consistently suggest that MDK is involved in the post-translational modification of C/EBPβ.

Mouse double minute 2 homolog (MDM2) is an E3 ubiquitin ligase, which targets C/EBPβ for degradation. Therefore, we hypothesized that MDK may prevent C/EBPβ degradation by impacting C/EBPβ-MDM2 complex. Co-IP assay showed that TGF-β treatment significantly hampered the interaction between C/EBPβ and MDM2. Contrarily, silencing MDK resulted in increased MDM2 binding to C/EBPβ (Fig. 5j). Furthermore, IF staining showed MDK interacted with C/EBPβ and remained in nucleus after 48 h of TGF-β treatment (Fig. 5k). In conclusion, we found that MDK accelerate EndMT via stabilizing C/EBPβ by disrupting C/EBPβ-MDM2 complex.

### MDK deficiency prevent renal fibrosis by EndMT in vivo

**Fig. 6:**
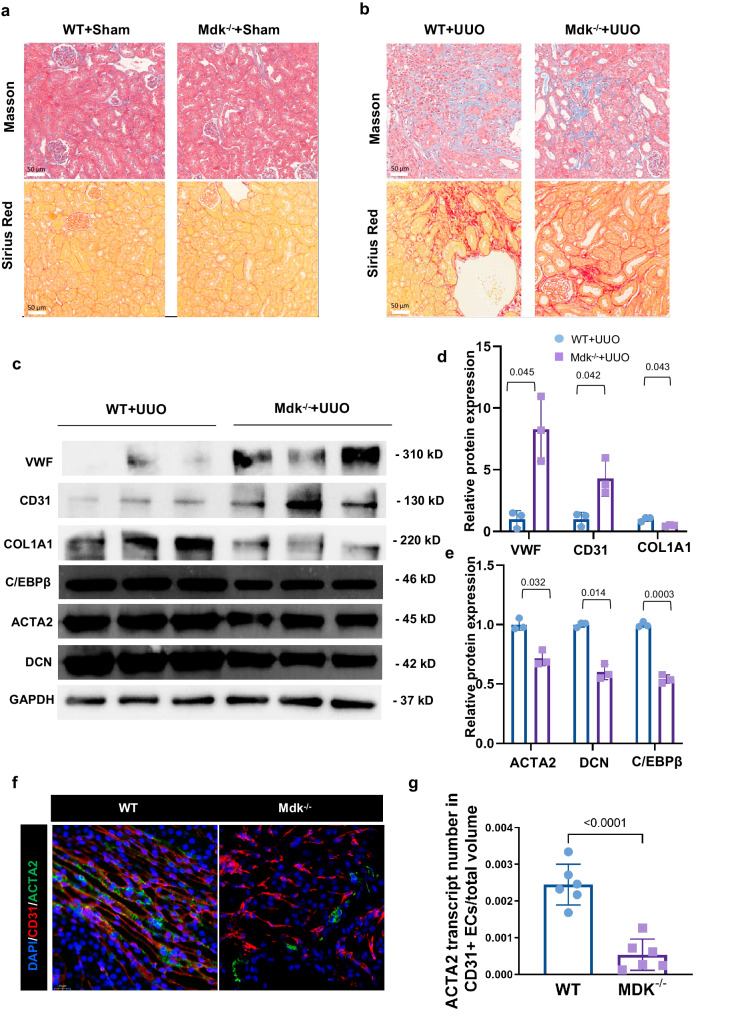
MDK deficiency prevent renal fibrosis by EndMT in vivo. **a**, **b** Masson staining and Sirius red staining of WT and Mdk^−/−^ mice subjected to UUO treatment. *n* = 6 for all groups. Original magnification, ×20. Scale bar, 50 μm. **c**–**e** WB analysis and quantification of ECM markers and ECs markers of WT and Mdk^−/−^ mice in UUO model. *n* = 3 for all groups. Each data point represents one animal. Data are presented as means ± SD (**f**, **g**) Representative image of CD31 and ACTA2 IF staining show CD31 + ACTA2+ ECs elevation in WT and Mdk^−/−^ mice in UUO model. *n* = 6 for all groups. Original magnification, ×40. Scale bar, 20 μm. Each data point represents one animal. All the error bars above denote standard deviation.

To study the functional relevance of MDK in renal fibrosis, we employed Mdk^−/−^ mice for further investigation. IF staining (Fig. S4a) and WB analysis (Fig. S4b) showed the knockout efficiency of Mdk in kidney from Mdk^−/−^ mice. WT mice had a higher fibrotic area than Mdk^−/−^ mice revealed by Masson’s trichrome staining and Sirius red staining (Fig. 6a, b). Moreover, knockout of Mdk reduced EndMT markers as determined by WB experiment (Fig. 6c–e) and decreased the number of CD31^+^ACTA2^+^ ECs in UUO mice (Fig. 6f, g). These results indicated that Mdk knockout can reduce the degree of renal fibrosis by inhibiting EndMT.

### MDK overexpression and deficiency in ECs alter renal fibrosis by mediating EndMT in vivo

**Fig. 7:**
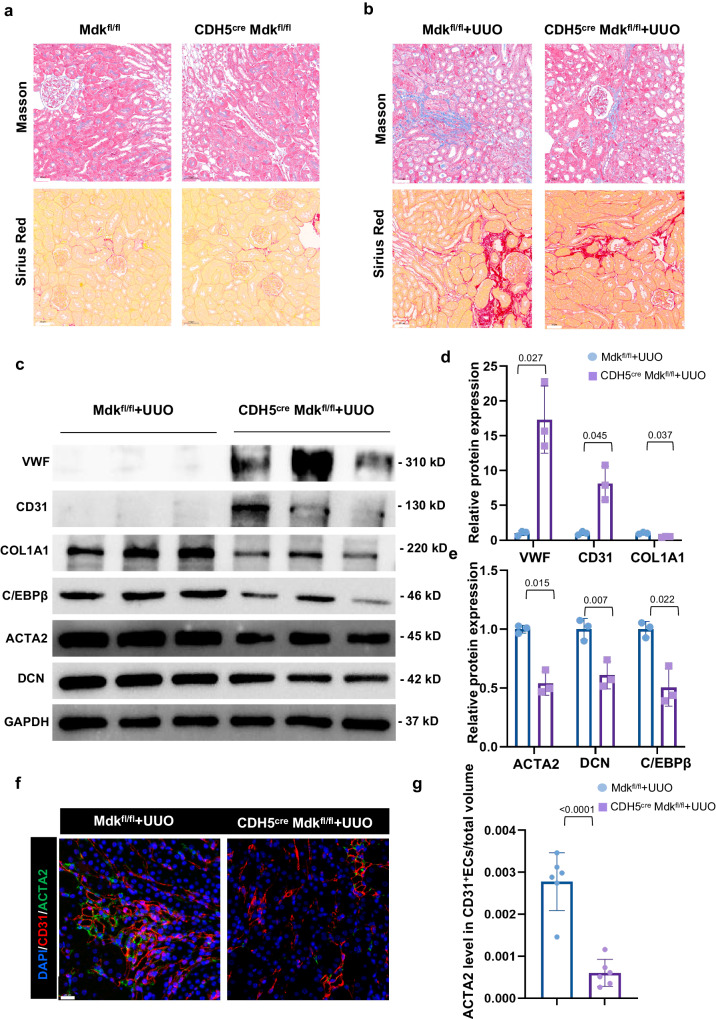
MDK deficiency in ECs ameliorates renal fibrosis by inhibiting EndMT in vivo. **a**, **b** Masson staining area and Sirius red staining of Mdk^fl/fl^ and CDH5^CRE^Mdk^fl/fl^ mice in UUO model. *n* = 6 for all groups. Original magnification, ×20. Scale bar, 50 μm. **c**–**e** WB analysis and quantification of ECM markers and ECs markers of Mdk^fl/fl^ and CDH5^CRE^Mdk^fl/fl^ mice in UUO model. *n* = 3 for all groups. Each data point represents one animal. Data are presented as means ± SD. **f**, **g** Representative image of CD31 and ACTA2 IF staining shows CD31 + ACTA2+ ECs elevation in Mdk^fl/fl^ and CDH5^CRE^Mdk^fl/fl^ mice in UUO model. *n* = 6 for all groups. Original magnification, ×40. Scale bar, 20 μm. Each data point represents one animal. All the error bars above denote standard deviation.

Since we found ECs have a predominant expression of midkine, we explored whether the loss of *Mdk* in ECs exerts a protective role in fibrosis. IF staining (Fig. S4c) and WB analysis (Fig. S4d) showed the absence of MDK in ECs. Masson’s trichrome staining and Sirius red staining revealed that there is no difference between two groups undergoing sham surgery, and the fibrotic area is more significant in *Mdk*^fl/fl^ + UUO group than CDH5^cre^ *Mdk*^fl/fl^ + UUO group (Fig. 7a, b). Moreover, the protein expressions of DCN, ACTA2, Col1A1, and C/EBPβ were higher in *Mdk*^fl/fl^ + UUO group. CDH5^cre^ *Mdk*^fl/fl^ + UUO mice had higher expressions of VWF and CD31 (Fig. 7c–e). We also performed co-staining of CD31 and ACTA2 to measure the EndMT, and found the *Mdk* deletion in ECs ameliorates fibrosis by suppressing EndMT (Fig. 7f, g).

**Fig. 8:**
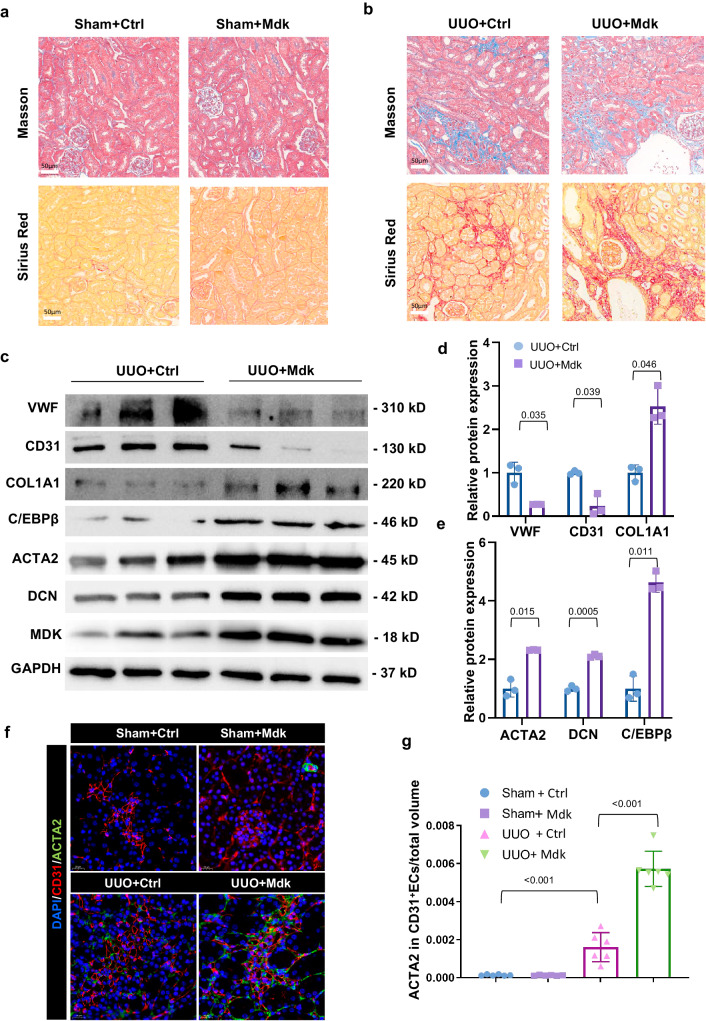
MDK overexpression in ECs deteriorates renal fibrosis by promoting EndMT in vivo. **a**, **b** Masson staining area and Sirius red staining of mice injected with or without AAVs in UUO model. *n* = 6 for all groups. Original magnification, ×20. Scale bar, 50 μm. **c**–**e** WB analysis and quantification of ECM markers and ECs markers of mice injected with or without AAVs in UUO model. *n* = 3 for all groups. Each data point represents one animal. Data are presented as means ± SD. **f**, **g** Representative image of CD31 and ACTA2 IF staining shows CD31 + ACTA2+ ECs elevation in mice injected with or without AAVs in UUO model. *n* = 6 for all groups. Original magnification, ×40. Scale bar, 20 μm. Each data point represents one animal. All the error bars above denote standard deviation.

Next, we want to specifically elucidate the roles of MDK expressed by ECs in EndMT of renal fibrosis. The endothelial-specific adeno-associated viruses (AAV) AAV-Ctrl (Ctrl) and AAV-Mdk (Mdk) were injected into UUO mice. Masson’s trichrome staining and Sirius red staining indicated that UUO mice exhibited renal fibrosis. Injection of Mdk promoted the progression of fibrosis in UUO mice (Fig. 8a, b). Mdk Injection increased EndMT markers levels and C/EBPβ protein levels, as detected by WB analysis (Fig. 8c–e) and IF staining (Fig. 8f, g).

## Discussion

We illustrate a new mechanism linking MDK and C/EBPβ in a fundamental TGF-β-induced EndMT process and renal fibrosis. Altogether, our study substantiates the involvement of MDK in mediating TGF-β-induced EndMT and renal fibrosis through stabilizing C/EBPβ, which is physiologically significant for emergence of CD31^+^ACTA2^+^ECs. We also demonstrate that blocking MDK inhibits EndMT and reverses renal fibrosis.

Fibroblasts release ECM which includes interstitial connective matrix and the basement membrane^24,25^. Collagen I and fibronectin are interstitial matrix which provide structural scaffolding for tissue. And collagen IV, laminins, and proteins compose the basement membrane which expresses various receptors to perform the function of signal transduction^26^. MMPs regulated by tissue inhibitors of TIMPs, prevent ECM emergence by cleaving peptide bonds of ECM proteins^27–29^. Excessive ECM production and unbalanced degradation results in renal fibrosis^30^. We collected the expression of COL1A1, COL4A1 and MMP9, TIMP2 in non-fibrotic or fibrotic patients to calculate ECM-related scores, measured the correlation among them by single-cell sequencing. Single-cell data for matching tissue can be achieved by deconvolving spatial RNA-seq transcriptome to predict cell position. And we found the co-location of ECs and MES, and the co-expression of their markers in spatial transcriptomics, Therefore, we proposed that ECs can transform into a mesenchymal phenotype to produce ECM. Inhibiting EndMT may serve as an effective therapy for delaying renal fibrosis.

Induction of the conversion of ECs into mesenchymal fibroblast-like cells promotes fibrosis progression^31,32^. We found CD31^+^ACTA2^+^ ECs as an intermediate state of EndMT in the study. A key advantage of our study lies in the samples of different severity to characterize the pathological stages of renal fibrosis. Pseudotime analysis of ECs during fibrosis showed that ECs downregulated the expression of FLT1, VWF, and gradually expressed mesenchymal markers ACTA2 and DCN. These ECs were going through an intermediate phase because they displayed some features of mesenchymal markers in limited levels and kept their original identity (the level of CD31 did not change significantly during the process which called partial EndMT).

Myofibroblasts are the main player involved in ECM production during fibrosis^33,34^. Multiple factors promote the EndMT of ECs, which contribute significantly to myofibroblasts emergence in renal fibrosis^35,36^. In our study, we believed MDK promoted the progression of partial EndMT or even accomplished a total ECs identity transition to myofibroblasts (CD31^−^ACTA2^+^). We evaluated the successful induction of partial EndMT using ACTA2 and CD31 since there is co-expression of both markers during conversion of ECs to mesenchymal-like myofibroblasts. Herein, we used TGF-β treated ECs to mimic EndMT in vitro. With reduction of endothelial markers and induction of ACTA2 expression, ECs gradually transformed into myofibroblasts, which secrete ECM, result in fibrosis. Therefore, blocking ACTA2 expression is a promising approach of inhibiting EndMT^37^. TGF-β improved ACTA2 and MDK in a dose- and time-dependent manner, suggesting that MDK may have a functional role in mediating ACTA2.

TGF-β is among the key mediator of EndMT through numerous pathways. EndMT was observed in the progression of renal fibrosis^38–40^. Nevertheless, the detailed molecular mechanism for the involvement of EndMT in kidney fibrosis is unknown. Weckbach et al. found targeting cytokine MDK attenuates inflammation and reduces fibrosis during experimental autoimmune myocarditis^41^. Studies also revealed that the tubulointerstitial damage, as well as macrophages infiltration, collagen deposition, and osteopontin level were strikingly milder in MDK-deficient mice. In addition to the proinflammatory state, the involvement of MDK in TGF-β induced EndMT is a highly plausible cause of fibrosis. The canonical/Smad -mediated and non canonical pathway of TGF-β signaling are seen as driver of EndMT. TGF-β controls gene expression through SMAD proteins, which are known signal transducers and transcriptional modulators. TGF-β contains two subfamilies, TGF-β subfamily and BMP subfamily. Smad2 and Smad3 respond to signaling by TGF-β subfamily and Smads 1,5 and 8 primarily by BMP subfamily. Canonical TGF-β signaling causes translocation of phosphorylated Smad2/3 complex into the nucleus and trigger the target gene expression. BMPs can also lead to Smad1/5/8 phosphorylation and translocation to nucleus. However, Xavier et al. found that partial depletion of TGF-βRII in ECs would reduced EndMT, and Smad2 signaling induced by TGF-β was also inhibited in ECs. Contrarily, Smad1/5 was unaffected^42^. Therefore, Canonical TGF-β signaling controls fibrosis and promotes gene expression by mobilizing Smad2 and Smad3 transcription factors^43^. There also existing other factors regulating MDK expression, such as P53. Meng et al. found the expression of MDK could be regulated by P53, which played an important role in gloma progression^44^. Combined with the essential roles of Smad2/3 both in EndMT and gene expression, we selected Smad3 in our further exploration. Bioinformatic analysis revealed that *Mdk* promoter region has a SMAD3-binding element, suggesting that TGF-β controls MDK expression by Smad2/Smad3 signaling. Luciferase assay further showed that SD-208, which blocks TGF-β-induced phosphorylation of SMAD2 and SMAD3, reversed the enhanced Mdk transcript activity by TGF-β. Moreover, WB analysis confirmed the upregulation of MDK could be reversed by knockdown of SMAD3. Therefore, we concluded that TGF-β/SMAD2/3 signaling mediates MDK expression, which may involve in EndMT process.

As an essential transcription factor, C/EBPβ is required for EMT in profibrotic process by binding to the ACTA2 promoter^45^. C/EBPβ activation also participated in pulmonary fibrosis^46^ and TGF-β-dependent fibroblast remodeling in asthma^47^. However, the role of C/EBPβ in EndMT of kidney is unclear. Thus, we reasoned that C/EBPβ acts as a key mediator in TGF-β-induced EndMT in ECs. SMAD proteins were accumulated in the nucleus upon TGF-β signaling was activated, leading to the activation of SMAD-C/EBPβ complex co-regulated promoters, while subsequently inhibiting other C/EBPβ-dependent transcription^48^. In this work, we observed increases of C/EBPβ in the same way as MDK, and knockdown of MDK reverse the upregulated level of C/EBPβ. Besides, C/EBPβ knockdown suppressed TGF-β induced EndMT. Based on Co-IP analysis, we concluded that MDK promotes EndMT by targeting C/EBPβ. We observed that MDK have no effect on mRNA level but do increase C/EBPβ protein stability. Therefore, these results indict that MDK functions as a regulator in TGF-β-induced EndMT by stabilizing C/EBPβ.

Cell death is related to ECs state^49,50^. MDK is also acknowledged as an anti-apoptosis factor. TGF-β triggers EMT but also induces apoptosis of cells that are going through EMT^51,52^. Similarly, there may be a dynamic balance in the relation between TGF-β-induced apoptosis of CD31^+^ACTA2^+^ ECs and TGF-β-induced emergence of CD31^+^ACTA2^+^ ECs. Therefore, the degree to which EC apoptosis contributes to EndMT is unclear, and whether TGF-β/MDK is involved in EC apoptosis is worthy of further study.

## Materials and methods

### Single cell isolation, library preparation, and sequencing

The kidney tissues from four patients were collected and washed with PBS three times. Three fibrosis tissues were obtained from a renal transplant patient who had biopsy-proven interstitial fibrosis. X1 was obtained from the area of fibrosis, X2 was obtained from the junction of non-fibrosis and fibrosis area, and X3 was taken from the non-fibrosis area. Three non-fibrosis tissues were collected from three patients (C1, C2, C3), respectively. The fibrotic tissues were obtained from a patient’s nonfunction kidney secondary to high-grade obstruction. The control samples were obtained from patients who received nephrectomy because of renal cancer without diabetes or chronic kidney disease. A total of 23,879 genes were obtained from 31,330 cells after single cell analysis of three non-fibrosis and three fibrosis samples. Of these, 51.23% (16,051 cells) were from the non-fibrosis group and 48.77% (15,279 cells) were from the fibrosis group.

The tissue was cut into ~1 mm^3^ pieces and incubated in the same dispase solution at 37 °C for 30 min. Then, the tissue was gently dissociated with a pipette and incubated in trypsin 0.05% solution diluted with PBS for 10 min. After the trypsin was deactivated with PBS, the samples were filtered out with a 70 mm filter. Single cells were counted with a hemocytometer. Kidney cells were preferentially sorted for single-cell sequencing.

The cellular suspension was loaded onto a Chromium Single Cell instrument (10x Genomics) to generate single-cell Gel Beads-in-emulsion (GEMs). Then, single-cell RNA sequence (scRNA) library was estimated by using version 1 Chromium Single-Cell 30 Library, Gel Bead & Multiplex Kit (10x Genomics). Sequencing was performed on the Illumina NextSeq6000, with a length of 150 bp. Cell Ranger (version 3.0.1) was used with default parameters to perform sample demultiplexing, barcode processing, and single-cell gene unique molecular index counting. All clinical procedures were approved by the Ethics Committee of Zhongshan Hospital, Fudan University (B2020-046R, B2020-050R). All ethical regulations relevant to human research participants were followed. All patients gave informed consent.

### Single-cell RNA sequence data processing

Data from 6 scRNA-Seq experiments (3 control, 3 fibrosis) were processed with Cell Ranger 3.0.1 and Seurat 4.1.1 in R (version 4.1.2). Cells with less than 200 unique molecular identifiers (UMIs) in a single cell or more than 10% of mitochondrion-derived UMI counts were considered low-quality cells and removed. Then, the influence of the UMI count and the percentage of mitochondrion-derived UMI counts were regressed out with the Scale Data function. Each data set was independently scaled and normalized and then merged to create a single-cell reference with Harmony. After defining clusters, the result was visualized by UMAP. Vinplot, Dotplot, and Featureplot were used to visualize the enriched expressing genes. Differentially expressed genes (DEGs) were detected by default Wilcoxon test and visualized by Heatmap. To construct a single-cell pseudotime trajectory and identify genes that change as cells undergo transition, the Monocle2 (v.2.4.0) algorithm was applied to cells. CellChat package was used to analyze the cell signaling pathway for its higher true positive rate, lower false positive rate, and higher accuracy compared with CellphoneDB and other packages^53^. Ggplot2 was used for data visualization.

### Spatial transcriptomics data analysis

Raw sequencing reads of spatial transcriptomics (ST) were quality checked and mapped using Space Ranger v1.1.0. Space Ranger aligns the barcodes in each read with a 55 μm spot coordinate relative to the fiducial frame, associating read counts with the image. After generating counts, Space Ranger clusters spots using a graph-based clustering algorithm where a nearest-neighbor network is built in a principal components space and a Louvain modularity optimization algorithm selects the modules of highly connected spots. The feature plots present expression levels that were normalized in Space Ranger. After mapping of the human kidney sample by Space Ranger, the data was processed in Seurat 4.1.1. The data was normalized by SCTransform and merged to build a unified UMAP. Genes enrichments were showed by SpatialFeaturePlot. To verify whether clusters identified in Spatial transcriptomics processing covered the right spot of kidney sample, FindAllMarkers was applied to identify DEGs between clusters and visualized by VolcanoPlot and DotPlot.

The scRNA-Seq data of X1 was transferred to spatial transcriptomic spots in humans by calculating prediction scores for each cell in every spot with Seurat (Code from: https://satijalab.org/seurat/articles/spatial_vignette.html)^54^. The spot was assigned to the cluster with the highest prediction score and mapped back to the spatial transcriptomic sample image. Then the AveragEexpression was used to calculate correlation between clusters identified in scRNA-seq and Spatial Transcriptome. The result was showed by ComplexHeatmap. To further analyze the relationship between endothelial and MES, co-location was performed and showed by SpatialFeaturePlot.

### Cell culture

Human umbilical vein endothelial cells (HUVECs) were purchased from Merck (SCCE001). They were cultured in 0.2% gelatin-coated culture flasks in endoGRO-LS complete culture media (SCME001, Merck, Burlington, MA, USA,) at 37 °C incubators with 5% CO_2_. We used early cell passage numbers (passages 3–7). For EndMT, HUVECs were treated with TGF-β in 2.5 ng/ml (Cat# 240-B-010, R&D SYSTEMS) for 48 h.

### Dual luciferase assay

The luciferase activity assay was performed according to the manufacturer’s protocol (MedChemExpress). The luciferase reporter gene constructs under the control of the promoter sequence of MDK with SMAD3 predicted seed match sites (AGTCCAGACC) and mutant sites were constructed by inserting DNA fragment into pGL3-basic vector.

### Chromatin immunoprecipitation (ChIP) -qPCR Assay

Then HUVECs treated with TGF-β (Cat# 240-B-010, R&D SYSTEMS) for 48 h were prepared for ChIP assay using ChIP assay kit (Cell Signaling Techbology, CST) according to the manufacturers’ protocol. A qPCR was subsequently performed. The primers were designed as previously reported^55^.

### Histologic analysis

The renal tissues were embedded in paraffin and sliced into 5 μm sections after 4, 7, and 10 days of UUO and sham operation. The sections were deparaffinized, rehydrated and stained with Sirius Red and Masson’s Trichrome. Renal fibrosis was assessed using ImageJ plugin (https://imagej.nih.gov). The positive area of Masson’s Trichrome was blue, and the positive area of Sirius Red was red.

### Antibodies and immunofluorescence (IF) staining

Kidney tissues were fixed in 4% formalin for 2 h at room temperature and frozen in paraffin after dehydration in 30% sucrose overnight. Cryosections of 5–10 μm were used to prepare slides, which were then blocked in 10% donkey serum. The primary antibody was incubated for 1 h, followed by three washes of 5 min each with PBS. The secondary antibodies were then incubated for 45 min. After staining with DAPI (1:10,000, Cell Signaling, Danvers, MA, USA), the slides were mounted with ProLong Gold (Cat# P10144, Invitrogen, Waltham, MA, USA) staining. The following antibodies were used: anti-CD31 (Cat# 28083-1-AP, 1:100, Proteintech, Rosemont, IL, USA), anti-ACTA2 (Cat# 14395-1-AP, 1:100, Proteintech), anti-MDK (Cat# 11009-1-AP, 1:100, Proteintech), Cy3 Goat anti-rabbit (1:300, Servicebio, Wuhan, China), Cy3 Goat anti-mouse (1:300, Servicebio), Cy5 Goat anti-rabbit (1:400, Servicebio), 488 Goat anti-RAbbit (1:300, Servicebio), HRP Goat anti-mouse (1:300, Servicebio), 488 Goat anti-mouse (1:300, Servicebio) and AF647 Goat anti-mouse (1:400, Servicebio). DAPI glows blue by UV excitation wavelength 330–380 nm and emission wavelength 420 nm; FITC glows green by excitation wavelength 465–495 nm and emission wavelength 515–555 nm; CY3 glows red by excitation wavelength 510–560 nm and emission wavelength 590 nm. AF647 glows pink by excitation wavelength 608–648 nm and emission wavelength 672–712 nm; and the 594 glows purplish red by excitation wavelength 594 nm and emission wavelength 615 nm.

### Western blot analysis

Samples were lysed on ice in RIPA buffer supplemented with protease inhibitors. Aliquots of cell lysates were boiled in SDS-PAGE sample buffer, fractionated on 12.5% SDS-PAGE gel and 10% SDS-PAGE, and transferred to PVDF membrane. The membranes were blocked with 5% BSA in TBST (Tris-buffered saline, 10 mM Tris-HCl [pH 7.5], 150 mM NaCl, and 0.1% Tween-20) for 1 h at room temperature and incubated with primary antibodies at 4 °C overnight. The primary antibodies were rabbit anti-VWF (1:1000, Cat# 11778-1-AP, Proteintech), rabbit anti-ACTA2 (1:1000, Cat# 14395-1-AP, Proteintech), rabbit anti-DCN (1:1000, Cat# A1669, ABclonal, Woburn, MA, USA), rabbit anti-β-actin (1:5000, Cat# 8457 S, Cell Signaling Technology), rabbit anti-GAPDH (1:1000, Cat# 2118 S, Cell Signaling Technology), rabbit C/EBPβ (1:1000, Cat# 43095 S, Cell Signaling Technology), rabbit anti-MDK (1:1000, Cat#11009-1-AP, Proteintech), rabbit Col1A1 (1:1000, Cat#72026, Cell Signaling Technology). The membranes were then incubated with horseradish peroxidase-conjugated secondary antibodies (1:2000, Cat# 7074 S, Cell Signaling Technology) for 1 h at room temperature. Visualization of the blots was performed using the standard protocol for electrochemiluminescence (ECL; Santa Cruz Biotechnology, Dallas, TX, USA). The relative intensity of the protein bands was quantified by digital densitometry using ImageJ software (NIH, Bethesda, MD, USA). β-actin was used as an internal standard in cytoplasm and lamin B1 was used as an internal standard in nucleus.

### Coimmunoprecipitation (Co-IP)

The experiment was performed according to the protocol from MedChemExpress (MedChemExpress, Monmouth Junction, NJ, USA). HUVEC lysates were collected and immunoprecipitated with anti-NCL antibody or anti-mouse IgG (Beyotime, Shanghai, China) in 4 °C for 4 h. The cell lysates were added with 20 μl protein A/G magnetic beads (MedChemExpress) and rotated at 4 °C overnight. After incubation overnight, the beads were washed three times with immunoprecipitation buffer. Immunoprecipitates were eluted by boiling with 1% (wt:vol) SDS sample buffer and boiled at 100 °C for 5 min followed by western blotting. Equal amounts of extracts were separated by SDS-PAGE, transferred onto nitrocellulose membranes, and blotted with specific Abs.

### Si-RNA transfection and endothelial-specific adeno-associated virus (AAV) injection

To knock down NCL and MDK expression in HUVEC, NCL and MDK siRNA (Ribobio, Guangzhou, China) mixed with RNAiMAX (Invitrogen) and Opti-MEM were transfected to HUVEC. siMDK-1, CCAAGAAAGGGAAGGGAAA, siMDK-2, GACCAAAGCAAAGGCCAAA. The endothelial-specific adeno-associated virus (AAV) was constructed according to previous study^56^. As reported, the AAV vector with Tie1-cre promoter would induce the expression of target gene expression in the endothlium.

### Animal model

Mdk^−/−^ mice, Mdk^flox/flox^ mice (Mdk^fl/fl^) and vascular endothelial-cadherin Cre recombinase-positive (CDH5-Cre) mice were purchased from Cyagen (Shanghai, China). Mdk^−/−^ mice were generated by CRISPR-Cas9 technology. Exon 1-exon5 of MDK-201(ENSMUST00000028672.12) transcript was used as the knockout region, which contains all the coding sequence. Endothelial-specific Mdk knockout mice were generated by crossing CDH5-Cre mice and Mdk^fl/fl^ mice with loxP sites flanking exon3 of Mdk gene. Male C57BL/6 mice were purchased from Cyagen (Shanghai, China). UUO surgery was performed on the left kidney of mice aged 8–12 weeks^57^. The ureter was obstructed by two-point ligations with silk sutures. Sham-operated mice underwent the same procedure, except for the obstruction of the left ureter. The incision was closed, and mice were allowed to recover. Mice were euthanized 4d, 7d, 10d after surgery. The kidneys were harvested for histology. To explore the effect of MDK in renal fibrosis, a total of 24 mice were divided into four groups: (1) Sham group +Ctrl: mice were subjected to sham operation and injeccted with empty adenovirus; (2) Sham + MDK group: mice were injected with MDK-inducing adenovirus; (3) UUO: mice were subjected to UUO surgery injected with empty adenovirus; (4) UUO + MDK: UUO mice were injected with injected with MDK-inducing adenovirus. MDK was injected through tail intravenous before model establishment. All animal experiments have obtained ethical approval from the Animal Ethics Committee of Zhongshan Hospital, Fudan University (2022-089).

### Statistics and reproducibility

Statistical significances were tested by independent sample t-test and one-way analysis of variance (ANOVA) with the Tukey multiple comparison test. *P* < 0.05 considered statistically significant. All data were analyzed using GraphPad Prism 9 software and presented as means ± standard deviation. The IF staining and western blot are representative. All experiments were performed at least three times.

### Supplementary information

Supplementary Information

Description of Additional Supplementary Files

Supplementary Data

reporting-summary

## Data Availability

The image in Fig. 1 were all created by the spatial and scRNA transcriptomics we performed. The raw data have been deposited in the Genome Sequence Archive^58^ in National Genomics Data Center^59^, China National Center for Bioinformation/Beijing Institute of Genomics, Chinese Academy of Sciences (GSA: HRA006794, HRA006796) that are publicly accessible at https://ngdc.cncb.ac.cn/gsa-human/browse/HRA006796; https://bigd.big.ac.cn/gsa-human/browse/HRA006794. The source data for the graphs in this study are provided in Supplementary Data. The WB uncropped images are provided in Supplementary Fig. 5 in Supplementary Information (Fig. S5). The predicted results of possible transcriptions factor of ACTA2 by online databate PROMO are provided in Supplementary Fig. 6 in Supplementary Information (Fig. S6). The authors declare that all other data supporting the findings of this study are available within the article and its Supplementary Information files, or are available from the authors upon request.
