# Supplementary material for: Midkine promotes renal fibrosis by stabilizing C/EBPβ to facilitate endothelial-mesenchymal transition
Source: Commun Biol. 2024 May 7;7:544. doi: 10.1038/s42003-024-06154-0 (PMC11076470; doi:10.1038/s42003-024-06154-0)
Supplement: Supplementary file 1 — Supplementary Information [file 42003_2024_6154_MOESM1_ESM.pdf]

Supplementary Figure 1

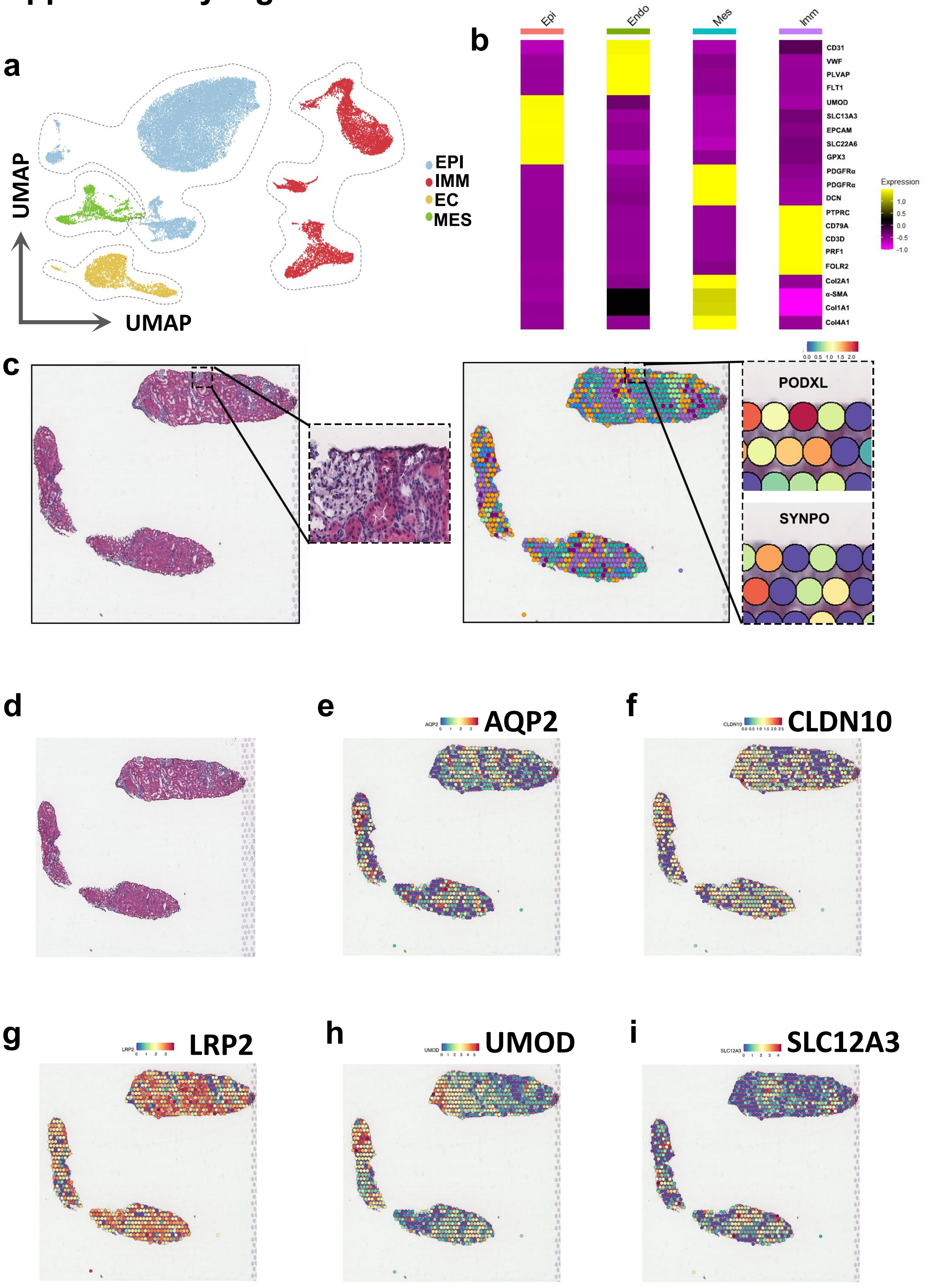

Figure S1 Spatial mapping and cell type identification of human kidney fibrosis tissue. (a) Unsupervised clustering of non-fibrosis (C1, C2, C3) and fibrosis patient (X1, X2, X3) demonstrates 4 cell types shown in the umap map. (b) Expression of relative markers used to classify clusters. (c-i) Expression levels of PODXL, SYNPO, AQP2, CLDN10, LRP2, UMOD and SLC12A3 in the spots over the high magnification region.

Supplementary Figure 2

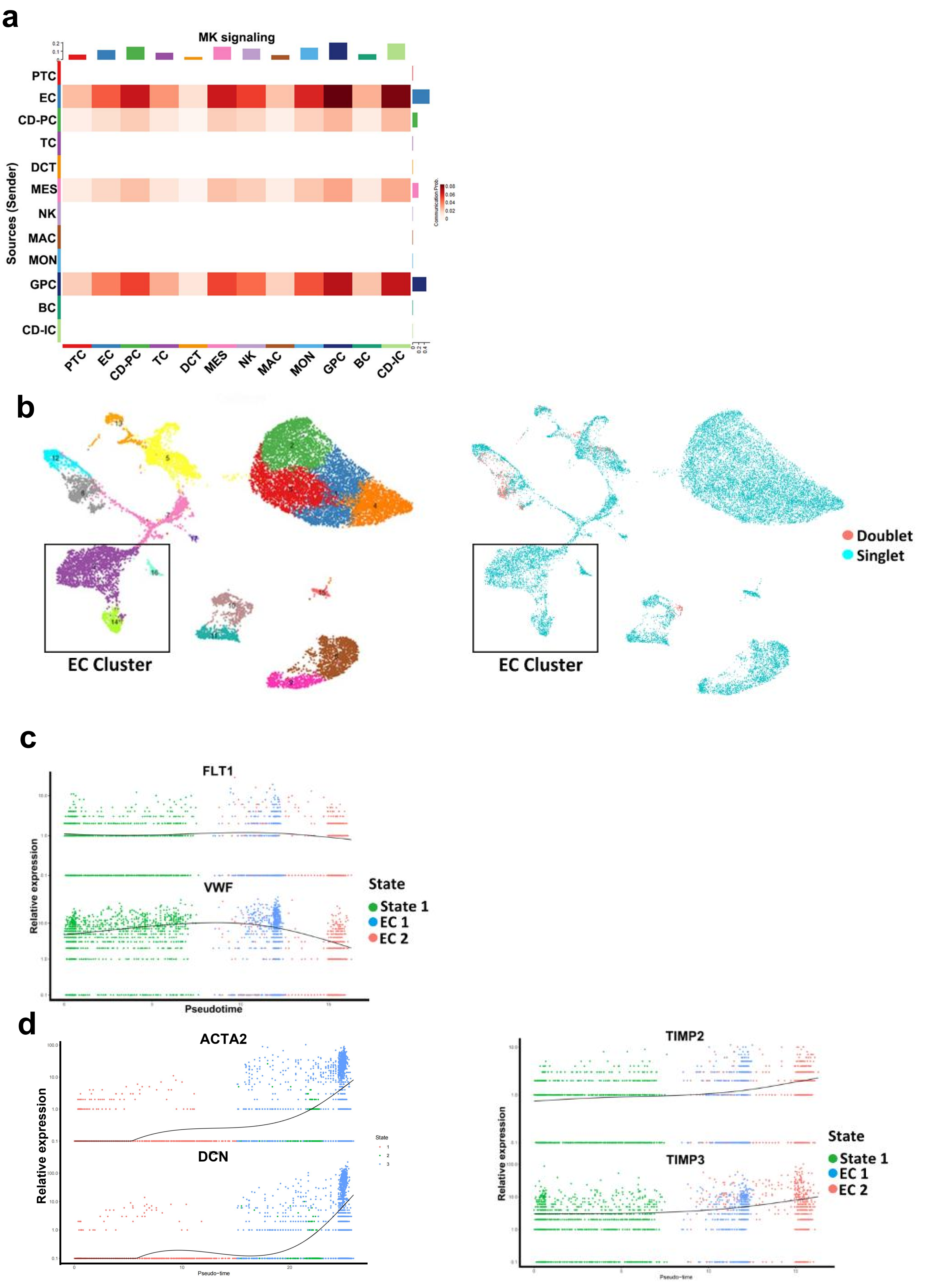

Figure S2 MDK effects during partial EndMT. (a) Heatmap of MK signaling roles in cell and cell communication. The communication probability of MK signaling is computed by summarizing the probabilities of its related ligand-receptor pairs. The darker the color, the greater the communication probability between the two cell types. (b) DoubletFinder shows rarely doublets were seen in EC clusters. (c) Pseudotime analysis shows the expressions of EC marker FLT1 and VWF decreased in EC2 state. (d) Pseudotime analysis shows the expressions of DCN, ACTA2, TIMP2 and TIMP2 increased in EC2 state.

Supplementary Figure 3

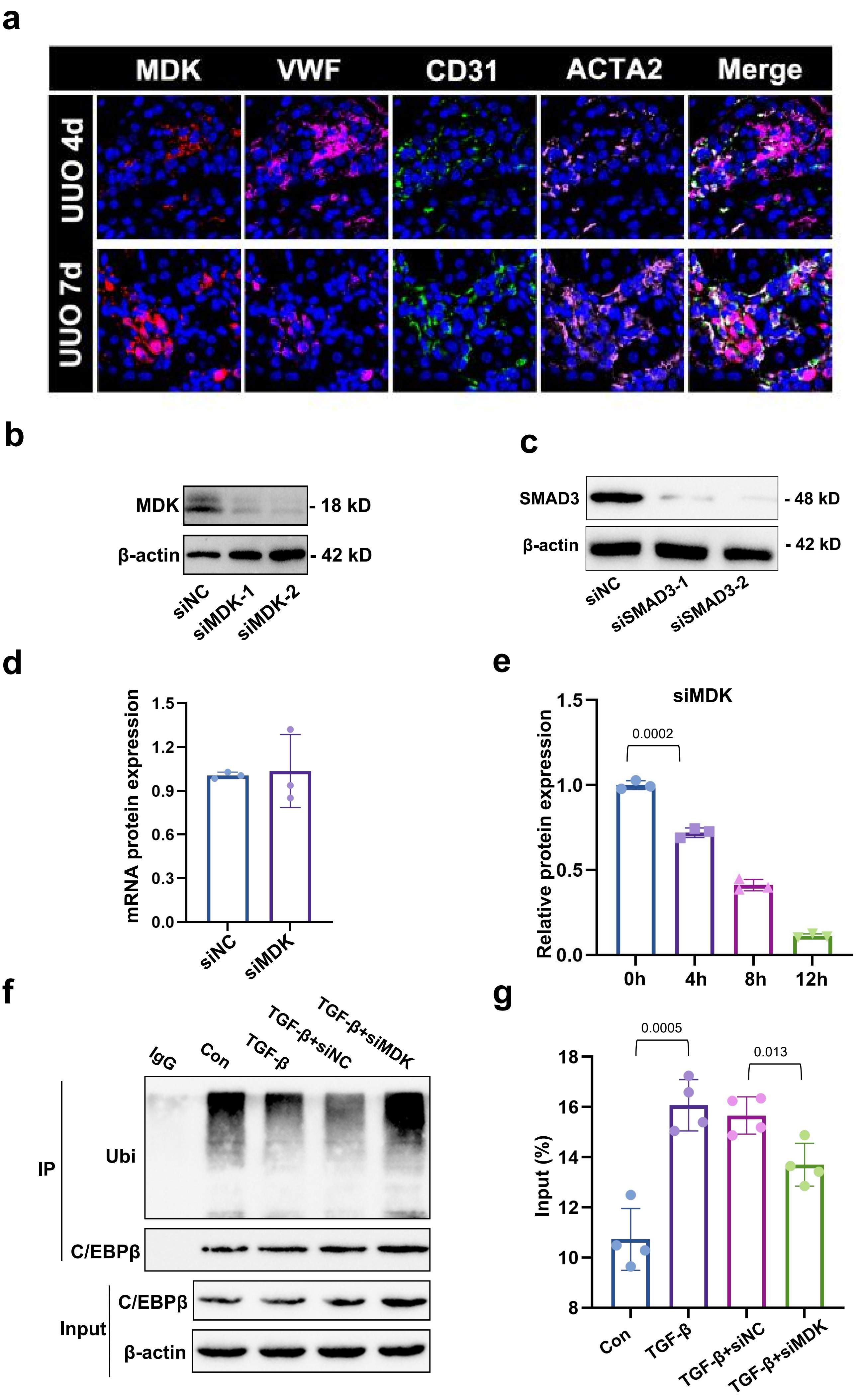

Figure S3 (a) Representative image of CD31, ACTA2, VWF and MDK IF staining shows ECs going through EndMT secret MDK. Original magnification, ×40. Scale, 20 μm. (b) The knockdown efficacy of MDK siRNA. (c) The knockdown efficacy of SMAD3 siRNA. (d) qPCR shows the effect of siMDK on C/EBPβ mRNA level. (e) Quantification of WB experiment of C/EBPβ in MDK knockdown cells after treatment with CHX for indicated times. (f) HUVECs lysates were immunoprecipitated with anti-C/EBPβ followed by immunoblotting with anti-ubiquitin and anti-C/EBPβ antibodies. The cells were pretreated with MG132 for 6h. (g) ChIP-qPCR shows the interaction between C/EBPβ and Acta2 promoter after MDK knockdown

Supplementary Figure 4

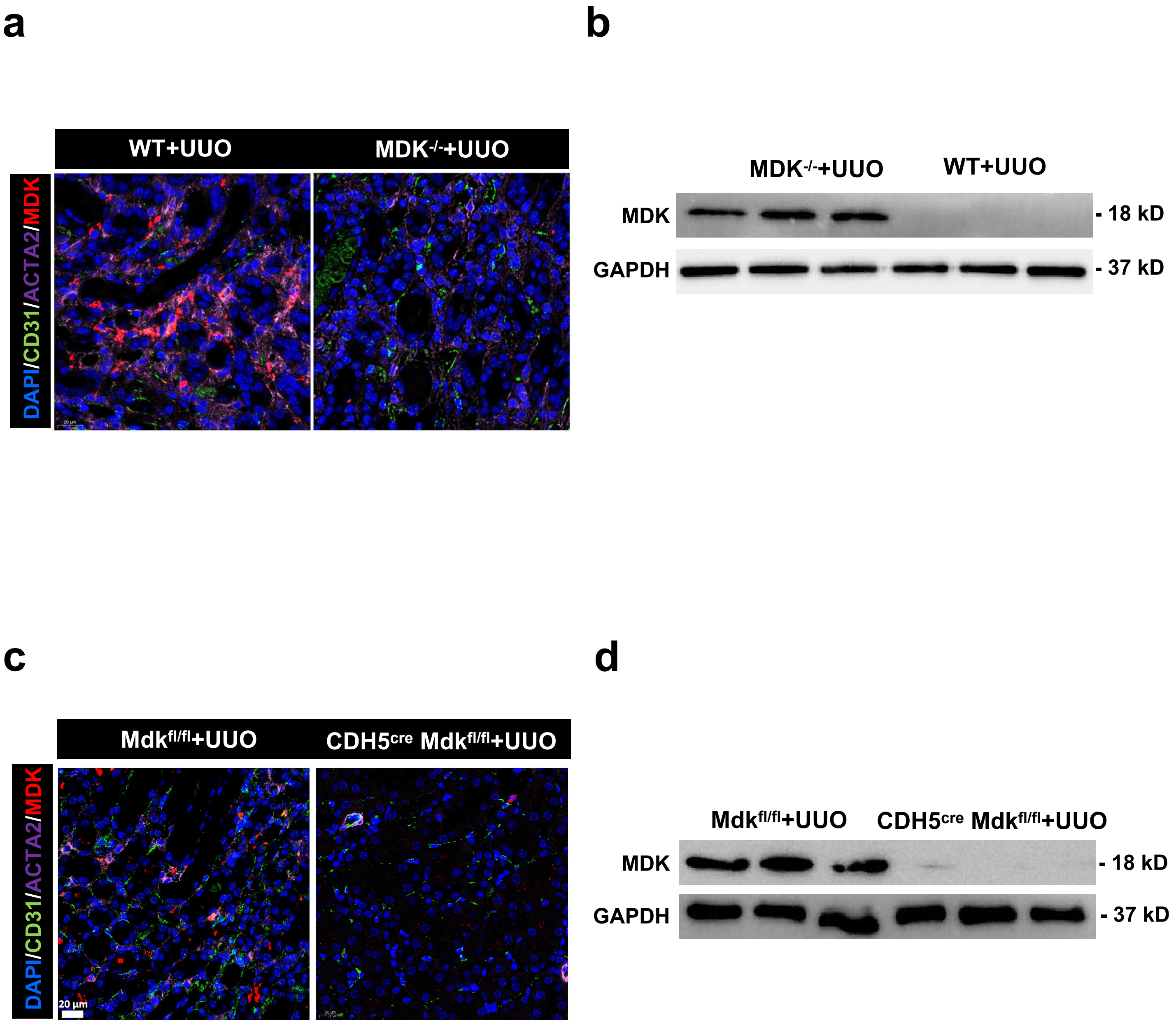

Figure S4 The knockout efficacy of MDK in different mice. (a, b) The knockout efficacy were testified by IF staining and WB experiment. (c, d) The knockout efficacy in ECs were testified by IF staining and WB experiment.

Supplementary Figure 5

Figure 3

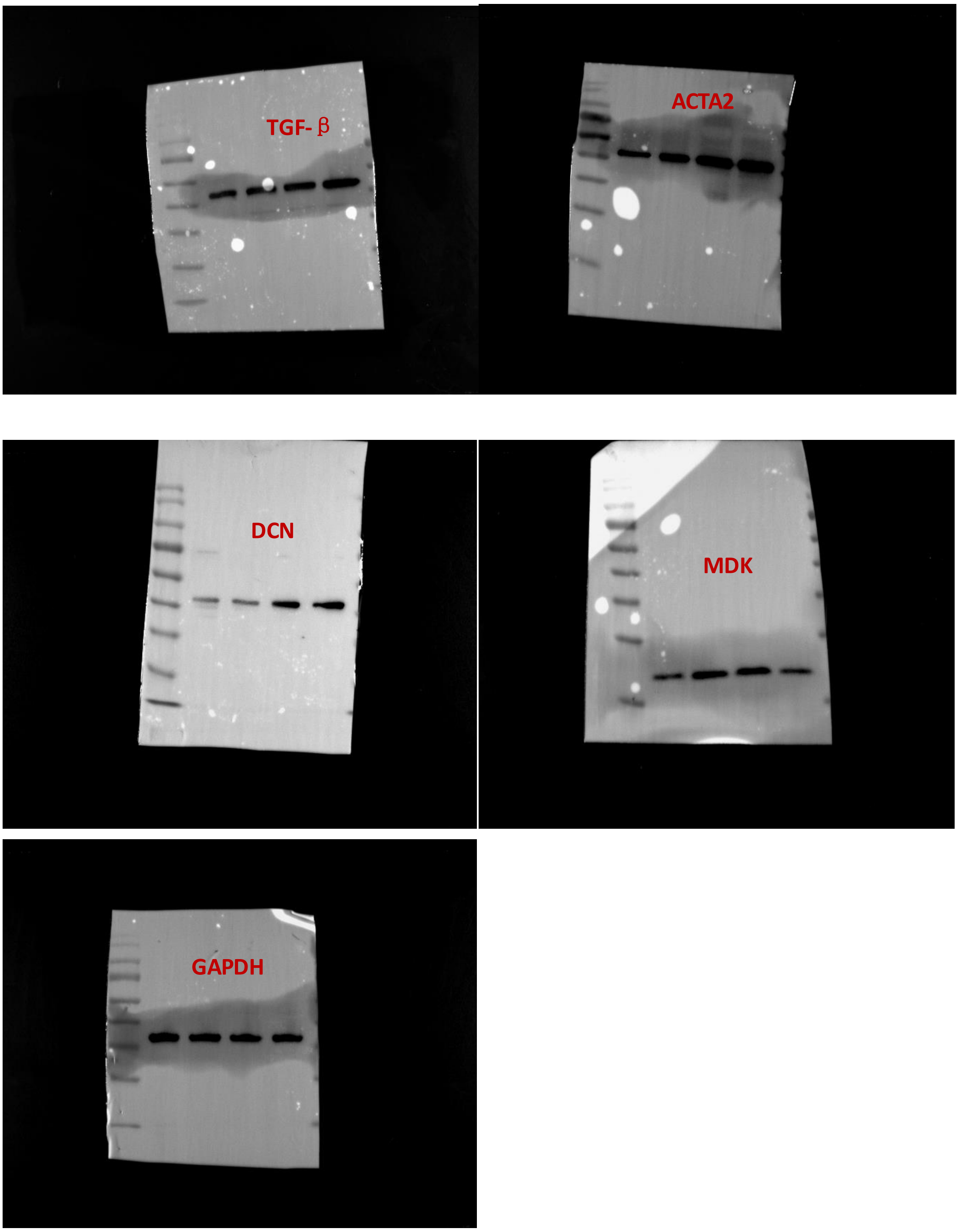

Figure 4a

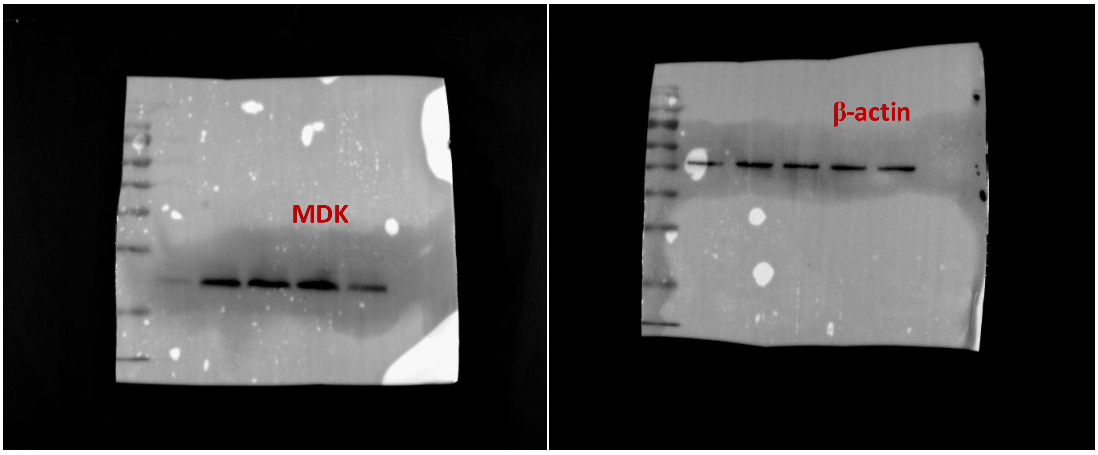

Figure 4b

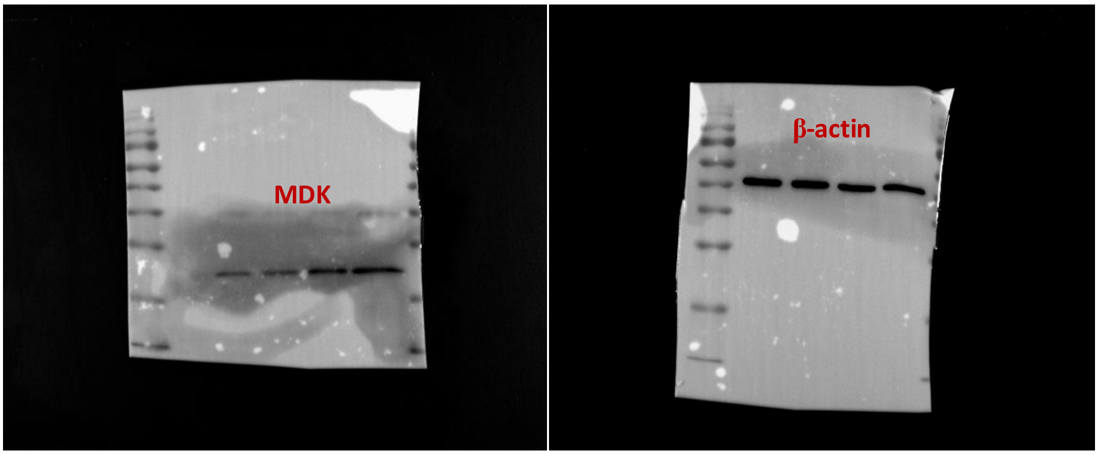

Figure 4c

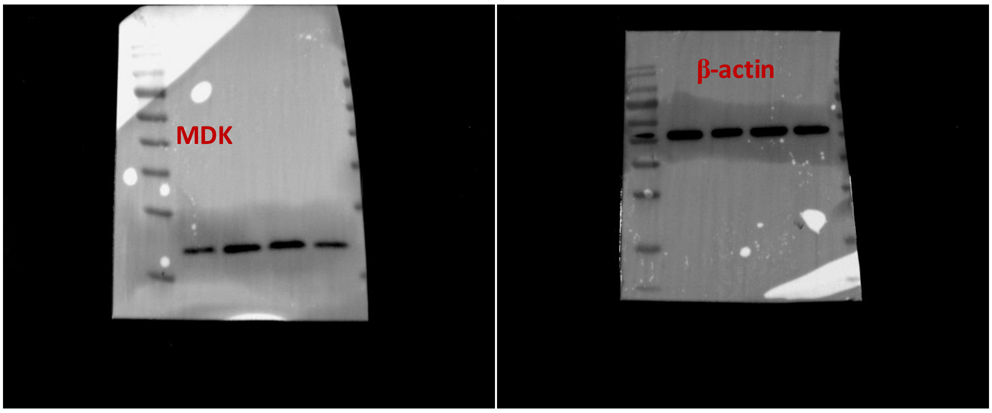

Figure 4f

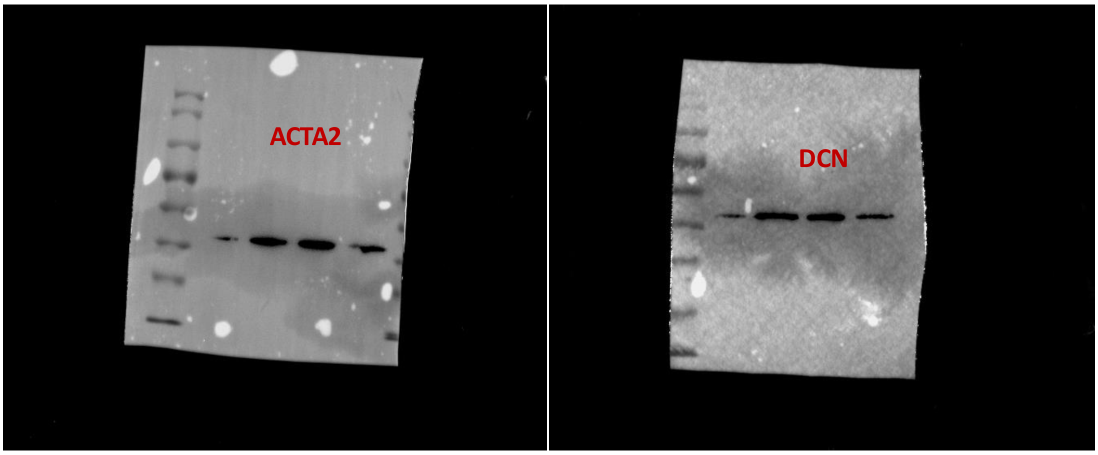

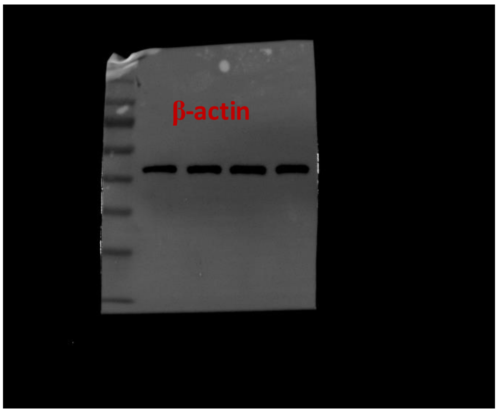

Figure 5a

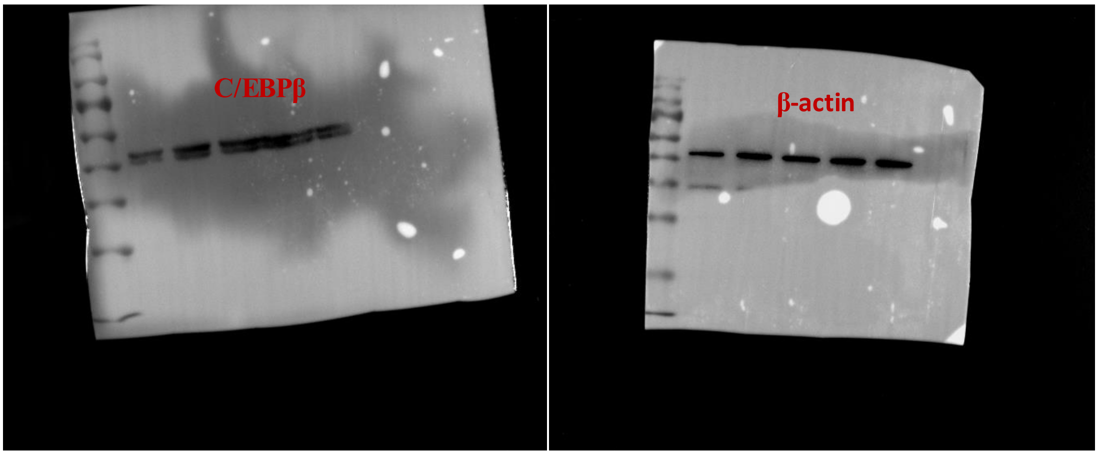

Figure 5b

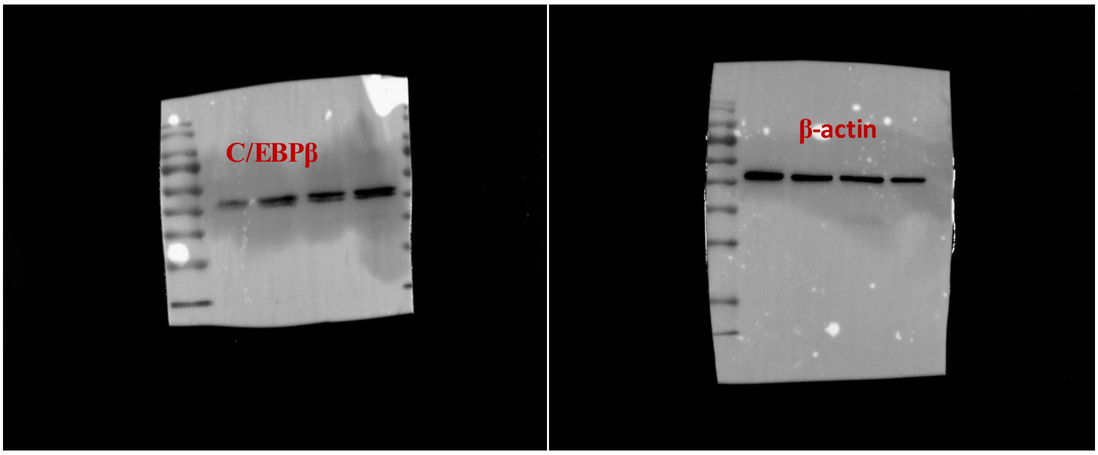

Figure 5c

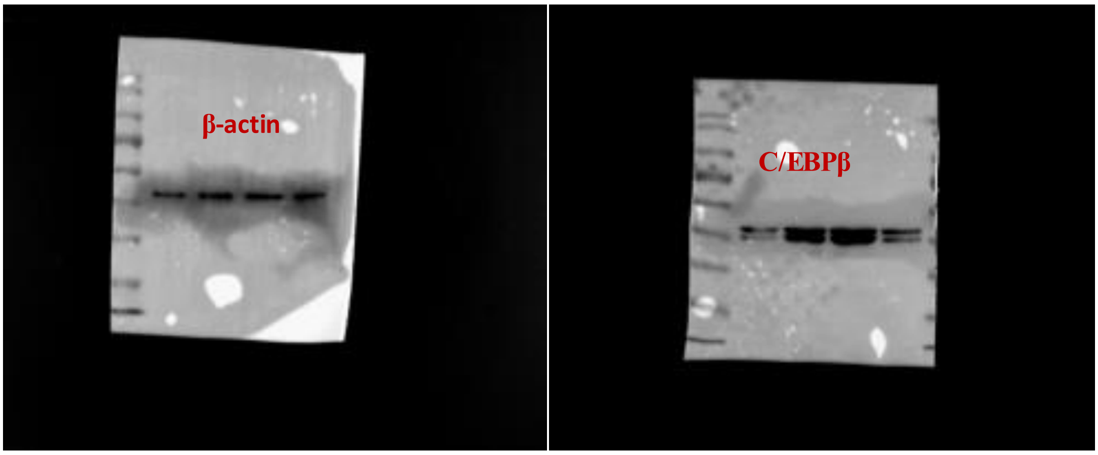

Figure 5d

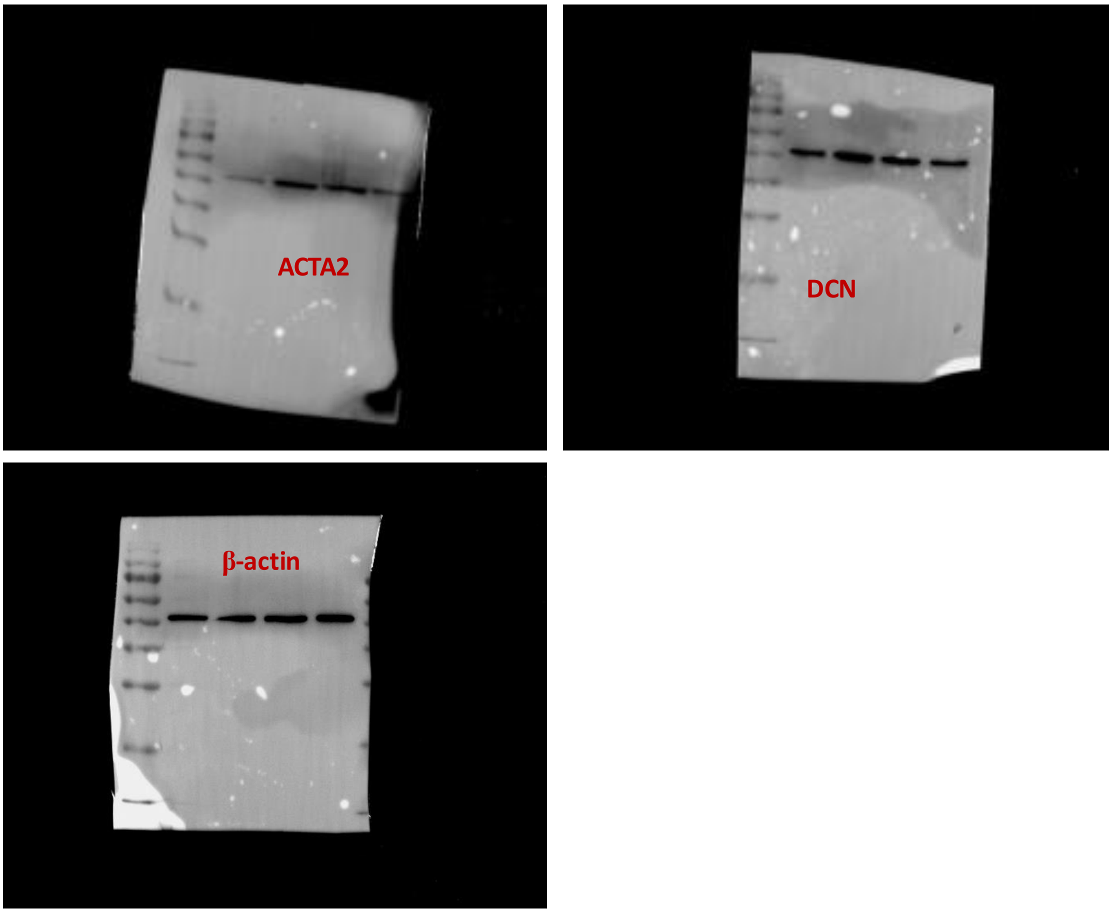

Figure 5e

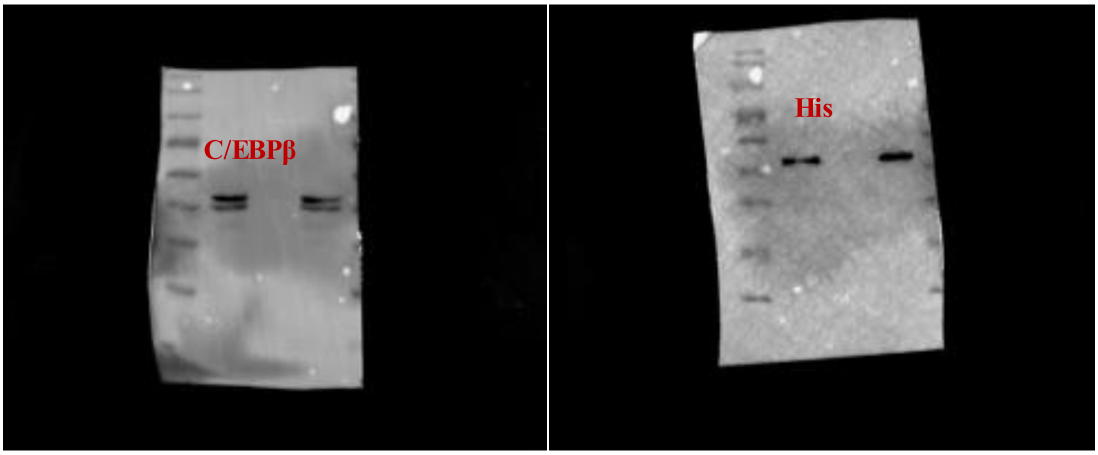

Figure 5f

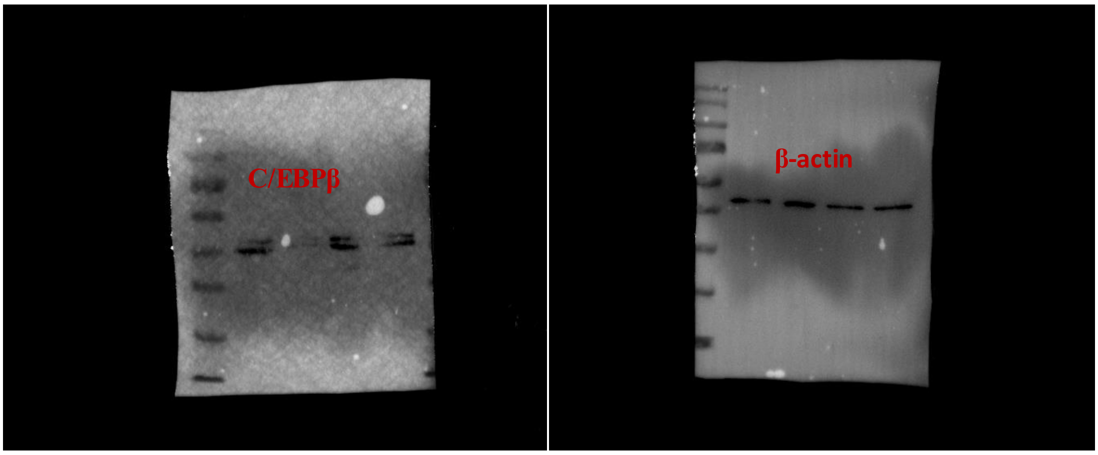

Figure 5h

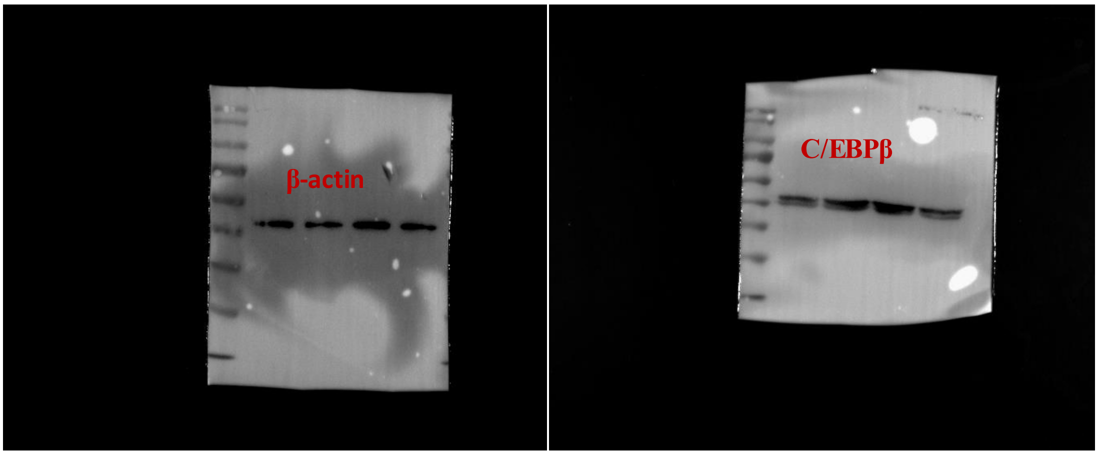

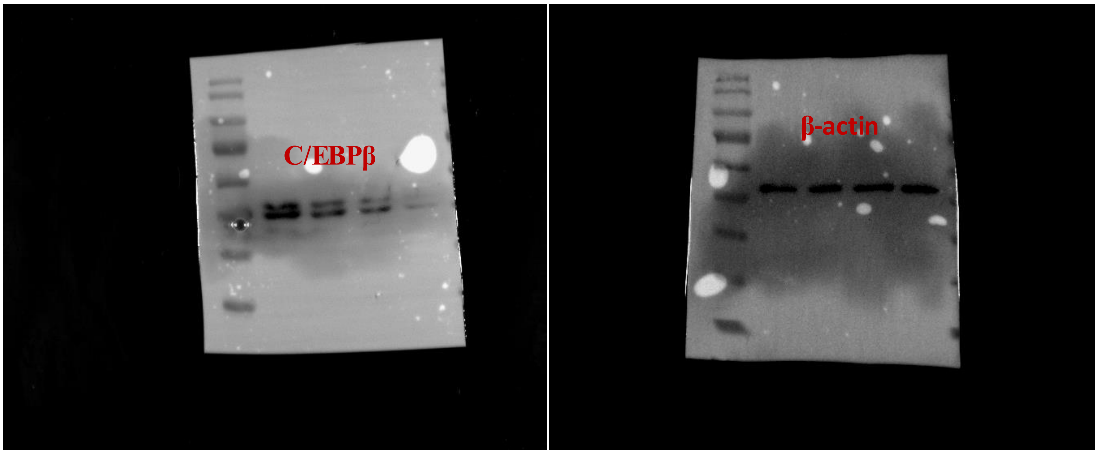

Figure 5j

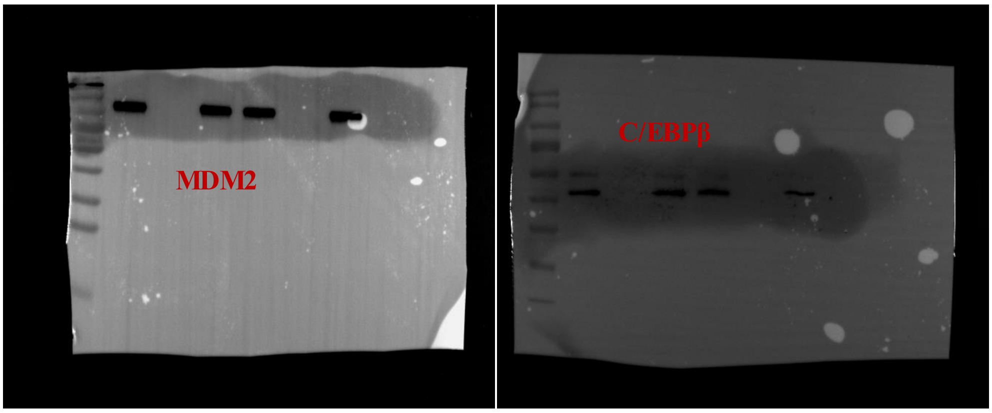

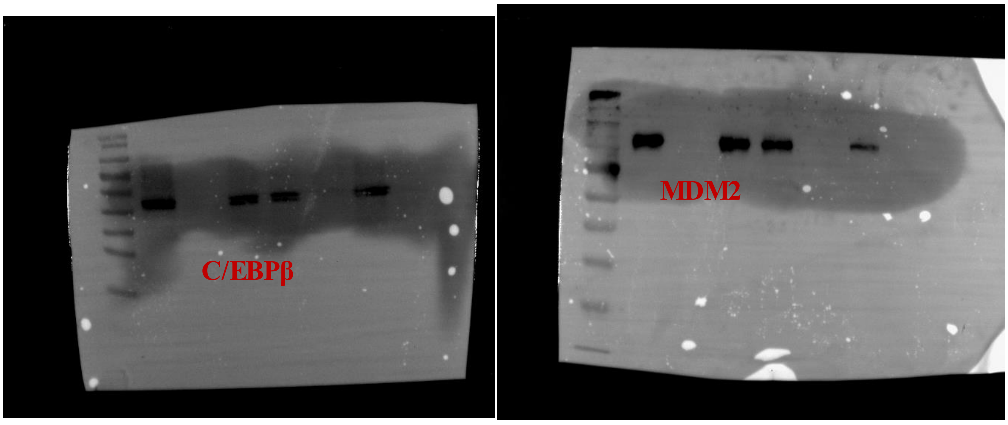

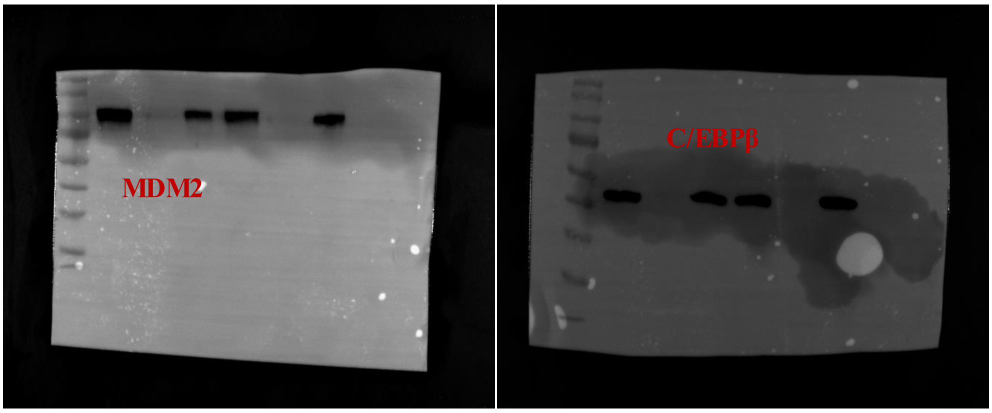

Figure 6c

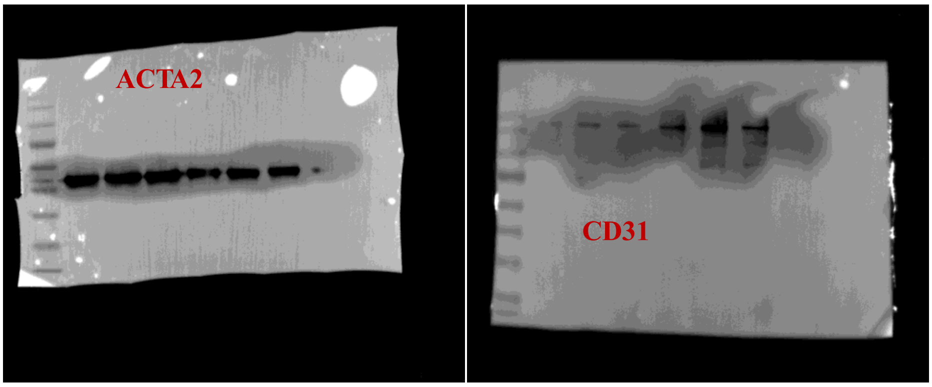

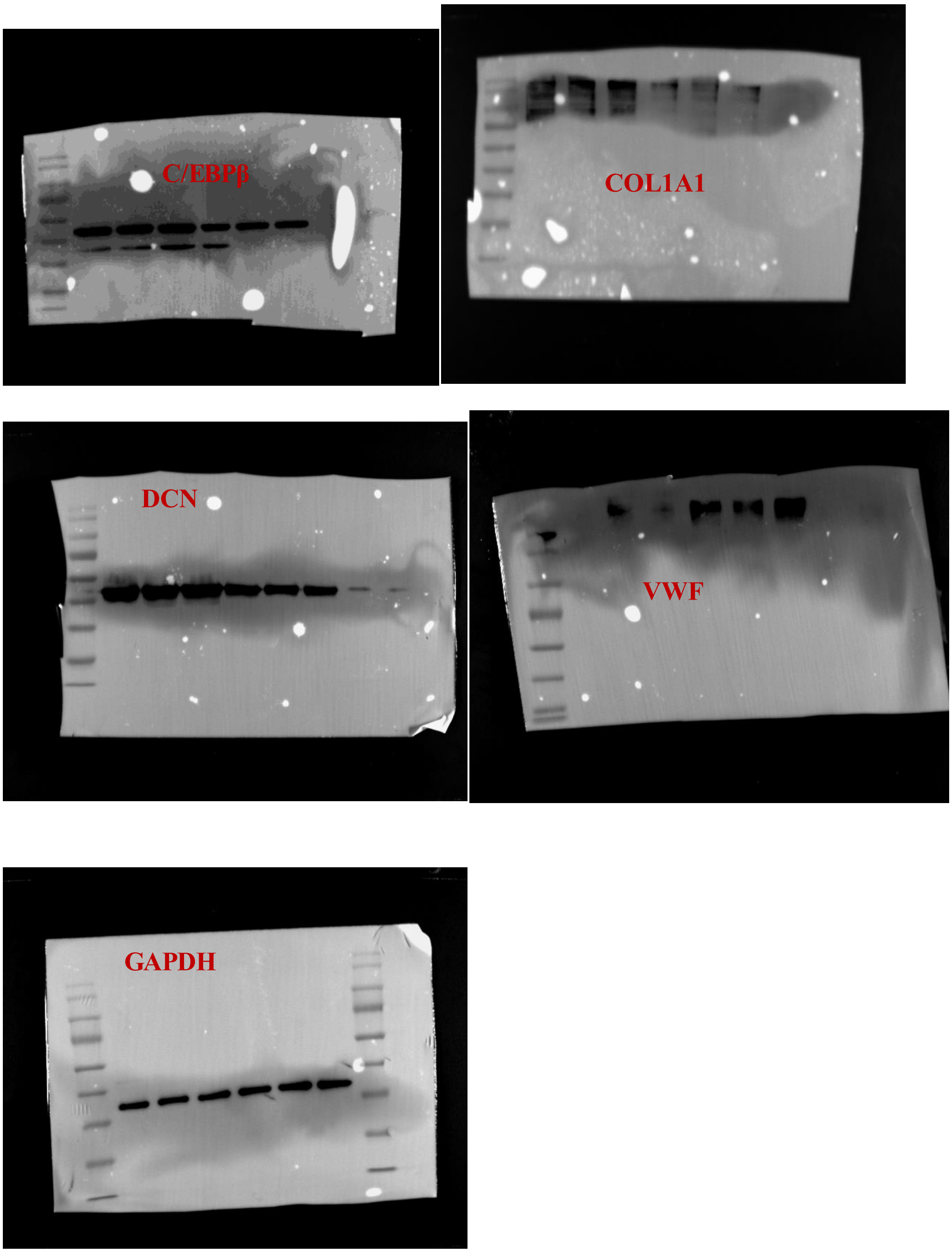

Figure 7c

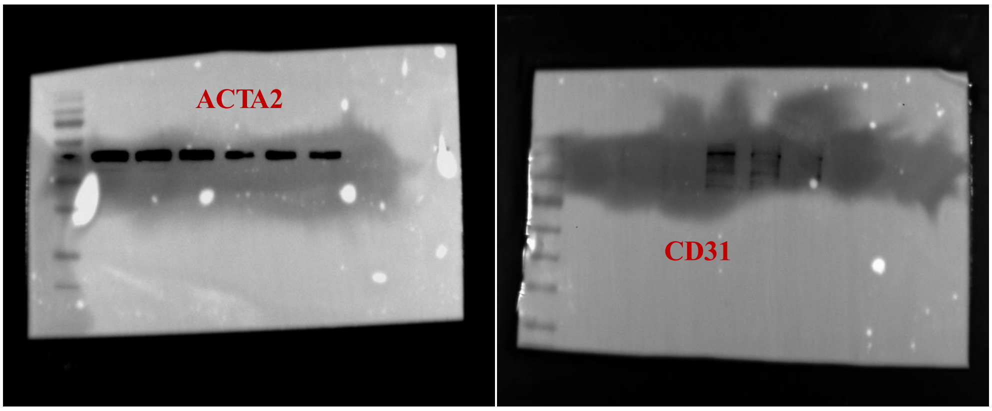

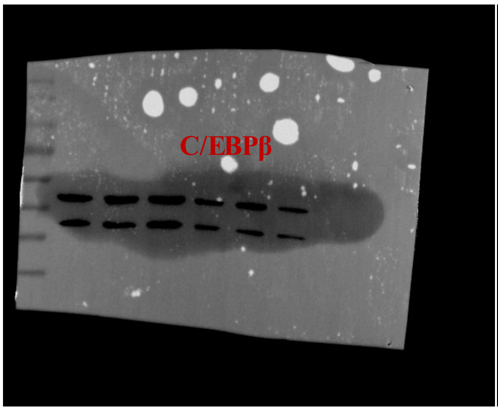

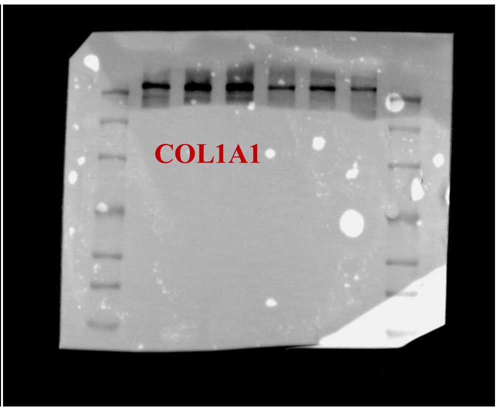

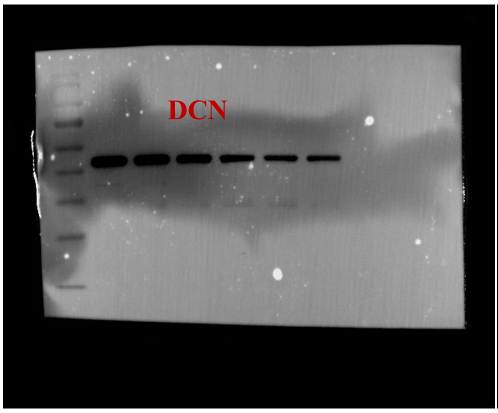

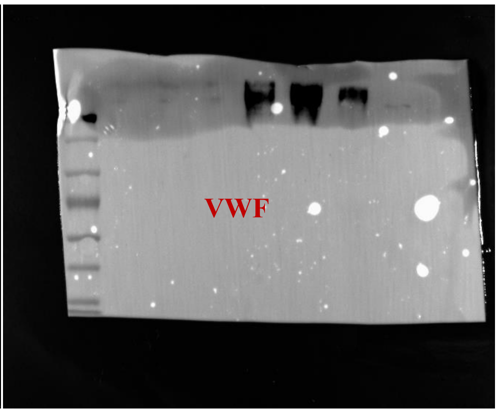

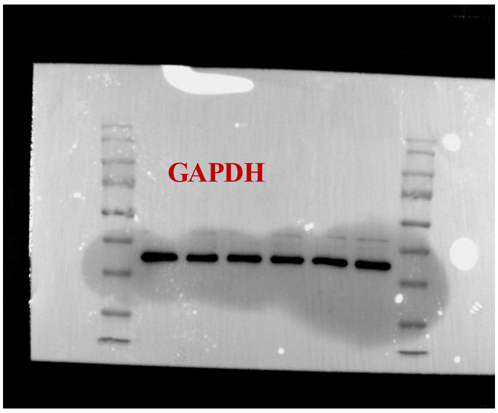

Figure 8c

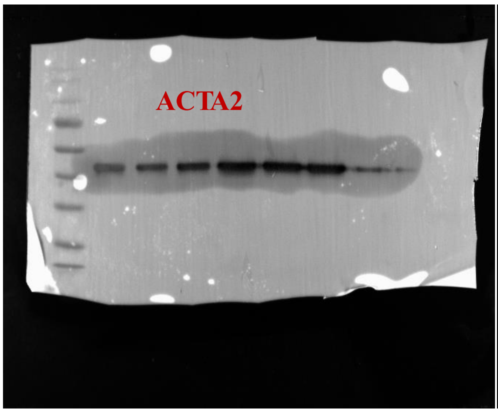

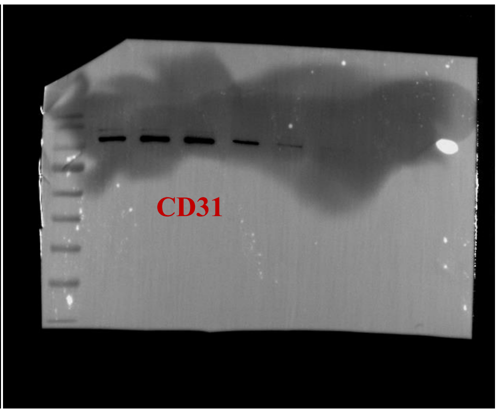

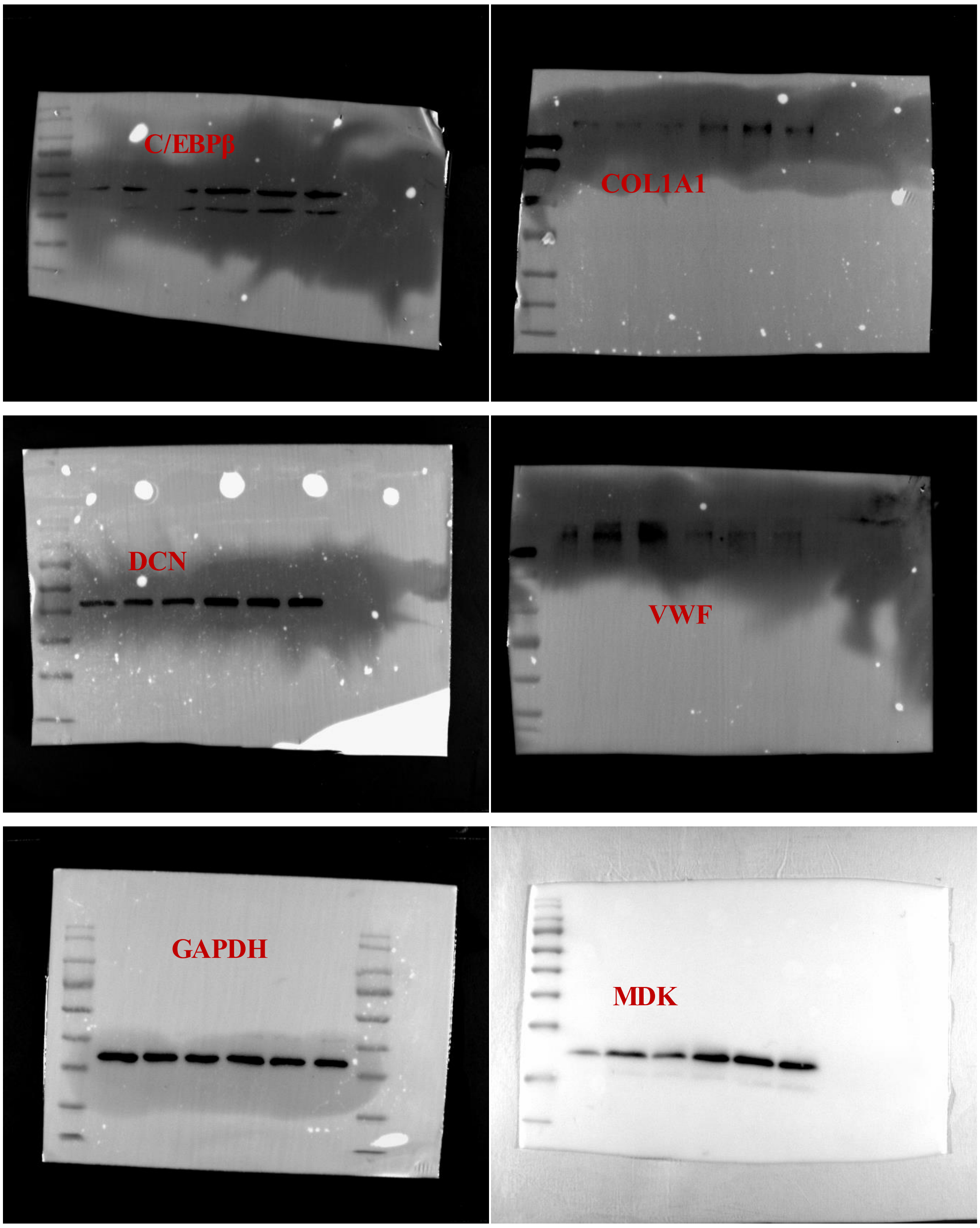
C/EBPβ
COL1A1
DCN
VWF
GAPDH
MDK

Figure S3b

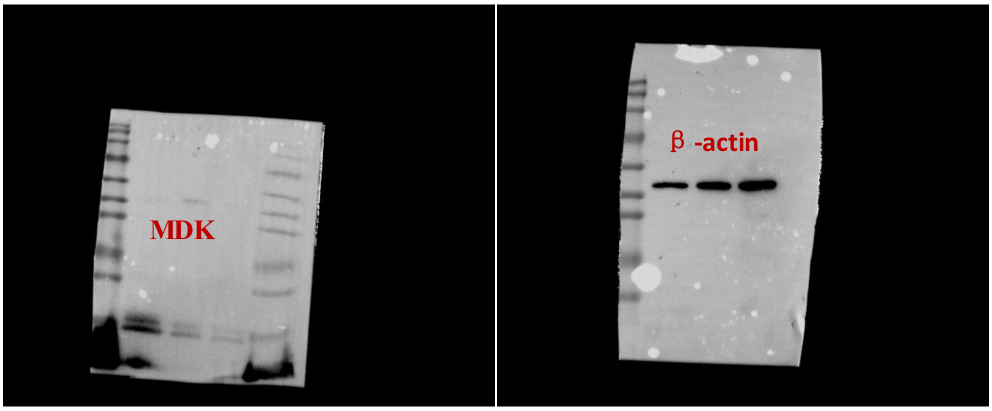

Figure S3c

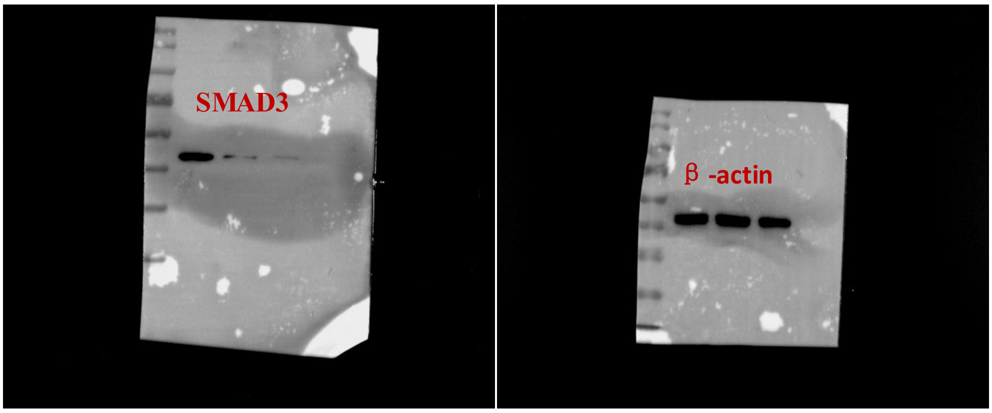

Figure S3e

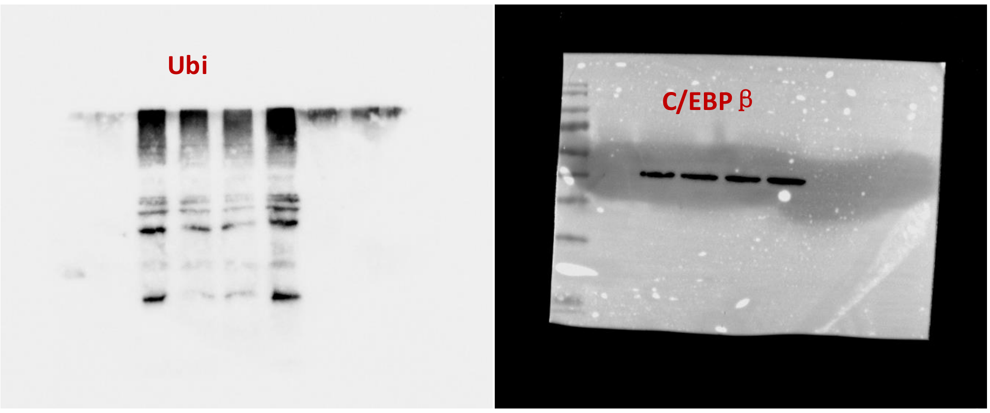

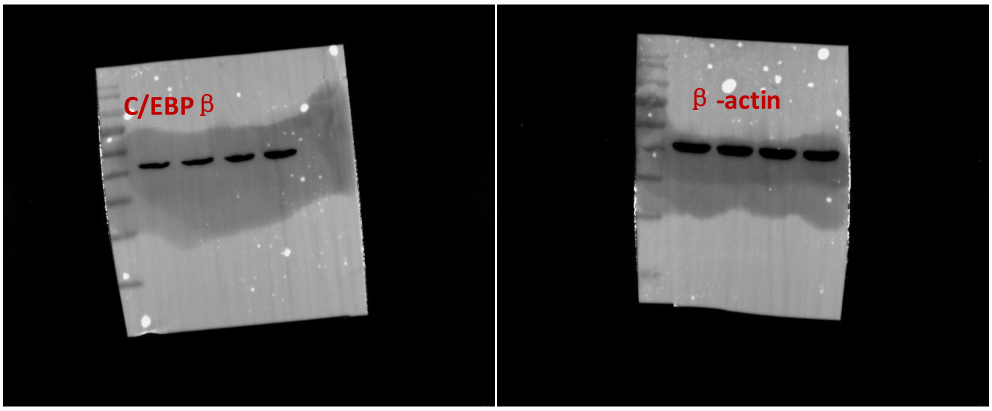

Figure S4b

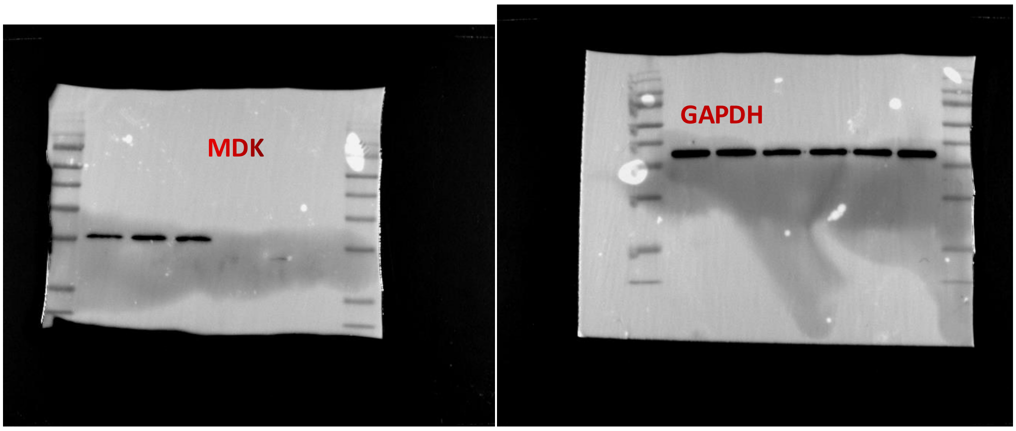

Figure S4d

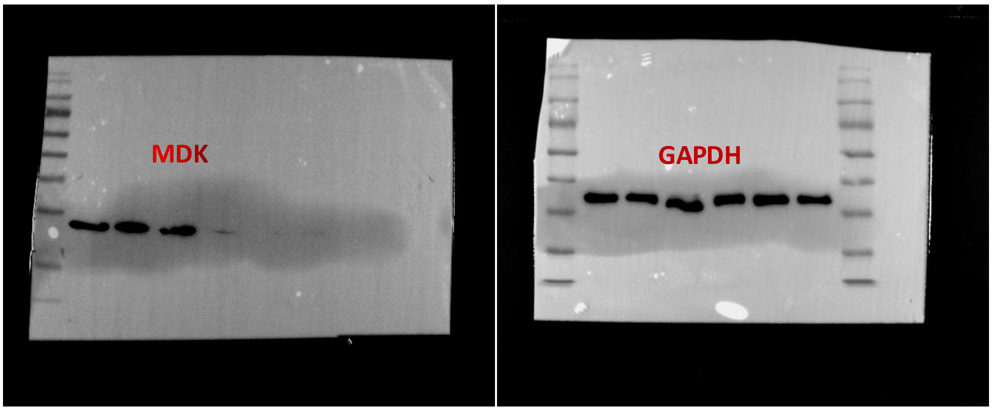

Supplementary Figure 6

Predicted results of possible transcription factor of ACTA2 by online database PROMO

```
-- Input sequence ---------------------------------------------------
>CCTGATCTCAGGTGATCCA
GCCCGCCTTGGCCTCCCAAAGTGCTGGGATTACAGGCGTGAGCCACTGCACCCGGCCTCTTTGACAGTTCTAAGAAGTAAAATAAACCAGAAGAAGGCAGGCAGAGAGTAATAGATATTGGGAACTGTTATTTTTCCTATTCTGATTTAAATTCCT
TTATTATGGAAAATTTCAAATGTATACAAAAGCAATGAAGATAATGAATGCCCGTAAACTCTCATCCAGCGTCATCGGTAATTAAGATTTTGCCCCATTTCAAATGCAGACATTTTTATATTTTCTTGCATAACTGCAATACCGTATTGCACTAAA
TGGAACTAACAGTCACTTGGATATTTTGGAAAGGATGGCTCAGAAAGGTATCTCTGAGGAGGTGATGTTCAGTCATGTAACTGATATTTACTAGTACCTACTGCATTCCAGACACTGCTTTAGGAGTTAAGGGTCCCTGAGTGAAGGACATTTGAG
CTAAGGTTTAAATGAAGTGAAGGGGCCAGGTGTGGTGGCTCATGCCAGTAATCCCAATGCTTTGGGAGGCCGAGGCGTGCGGATCGTTTGAGCCCAAGTTCGACACCAGCCTCAGCAACATAGTGAGACCGTGTCTCTAAAAAGAATAACAAATTA
AAAAGAATAAATAGGCCAGATGCAGTGGCTCAGGTCTGTAATACCAGCACTTTGGGAGGCTGAGGTGGGCGGATCACAAGGTCAGGAGTTCGAGACCAGCCTGGCCAACATGGTGAAACCCTGTCTCTACTAAAAAGACAAAAAAATTAGCCGGGC
ATGGTGGCACACGCCTGTAATCCCAGCTACTCGGAAGGCTGAGGCAGGAGAATCGCTTGAACCTGGGAGGCGGAGGTTGCAGTGAGCCGAGATCATGCCACTGCACTCCAGCCTGGCAGACAGAGTGAGACTCTGTCTCAAAAAAAAAAAAAAAAA
AAAATAGAAAAAAAAAGAAGAAATGAAGTGAAGGAACAAGCTGGAGTGGGTATCTGTGGGACTAGCAAGGCAGGCAGAGGGAACAGC
AGATGCAGGAGCCCCGAAATAAGACTGTCTGAGGAACAGACAGGACGCCAGTGTGGCTGGAGTGGAGTAGGCGTGAGAGAGGGAGTTGAGATCAGCCAGATTTGATAGCACCTTGTGGCTCACGGTGAGGACTTGGGCATTTGCCGTGAGATGGAG
CCAGGTTCAGAGCAGAGGAGTGACAAGACAATTTATAGCATGCCCATGGAGGGCAGAGATTTCTGTCTCTTTTTTAAAAAAATTGAAAAAAAATTTTGTAGAGACAGGGTCTTGCACTTTGTTGTCCAGGCTGGCATGCAGTGGTGTTATCATAGT
TCACTGCAACCTCGAACTCCTCGACTCCAGCGATCCTCCCACTTCATCCTCCCAAAGTGCTGGGATTAAAGGTGTGAGCCACCATGCCTAGCCTATTTCTATCTGTTTCATTTGTCCTCAGTCCACAGTAGATCCCAGCCCAGCACACAGTAGTAG
TTCAATAAACATTTGTTGCACAAATAGAGCAGATCAGTTTACATGGAGCTGTGTTATTTTGTATGTTCCAGGGTGTGGCATGCCATGATTTATTTAGCCCCCCCGTGGATGGTCATCTGGCTTCTTACAGGCTTGTCTTAAGCATTGCGTGAAATT
AATTATTACATTGCTCTTAGCACTGGAGGAAGTGCTTAATCTGTGTTAGTGATTATCATGACTATTTGTGTTGTTATTAACACAGTGGGTGCAAGGGAGACCCAGATGGAGATAGGGCTGGGGGGGCAACCTAGGGTGACACACGCACCTGGGGAG
GAGGGGCATGTGGCTTCTATGGTGGTAGCCCCTCCCTGCCCCTGACGCGTCTCTCCTGCCTGCAGGTACGTCTGGCCCACCGGGCTACGAGATGCGCTTGGGGGGAGCCAGGACGGAGGAAGAGGAGAGAGAAAGAGAAGTAAAGTCAGAGAGGTG
AGTTGGCAGGATGGGGAGAAAGAGAGGGATGGGGTGGGGAGGGGAATGAATAAAGAGATGGGGAGAGAGGCAGGAAGCTAGAGAGGG
GCTCTGAGCAGGGGCCAGAGGGAGATGAGCTATGAAGACCCACAGAGTGAAGTAACAGAGGGATGGGGGTGAAGGGGAGAAGAGAGACAGGGAGATGGAAGGAAAAACGCAGAAATGGAGAGACAAAATGAGAGAGACAGATACAGACACAGAGTT
AGGCCAAGGAGAGACAAAGACAGATACACAACAAGGCAAGAGGCGAAGATGAGGAGGGACAGAGACTGAGAAAGAAAATCAGGCGGGCGCGGCGGCTCACGATGGTAATACCAACACTTTGGGACGCTGAAGCAGGAGGATCGCTTGAGCCCAGGA
GTTCGAGAGTAGCCCAGGCAGCAGACTGAGATCCCATCTCTACCAAAAAAAAAAAAAAAAAAAAAAAAAAAAAGCTAGGAGTGGTGGCGCTTGCCTGTGGTTGGAGCTACTCCGGAGGCTGAGGCGGGAGGATGGCTTGCGCTCAGGAGGTTGAGGCT
GCAGCGAGCCATGATCGTGCCACTATACTCCAGCCTGGGTGGCAGAGCGAGACCCAGTCTCAAAACAAAAAGAAAATCAGACAGGTGGGGAGAGACAGAATAAGATAGGATGTTAGAAGATAAGAGAGACCGAATTGGAGATGGAAGAGGGGATG
CGGGGAGAGACGAAGTGAGAGAGGCTGGCGCGGTAGCGGGTGGGGGATGGGGCAGTGGAGGGCCGTTTTCCTCCCTCCACGAGCCCTGAGCCCTGACCCCGCCCGCCGCCCGCAG

-- Factors predicted by PROMO in this sequence ----------------------
NAME; MATRIX_WIDTH;
C/EBPbeta [T00581]; 4
NF-1 [T00539]; 8
ENKTF-1 [T00255]; 8
TFIID [T00820]; 7
SRY [T00997]; 9
TCF-4E [T02878]; 7
NFI/CTF [T00094]; 8
GR [T05076]; 7
GR-alpha [T00337]; 5
AP-2alphaA [T00035]; 6
RXR-alpha [T01345]; 7
LEF-1 [T02905]; 8
TCF-4 [T02918]; 10
MEF-2A [T01005]; 11
HNF-3alpha [T02512]; 8
GR-beta [T01920]; 5
FOXP3 [T04280]; 6
C/EBPalpha [T00105]; 7
NF-Y [T00150]; 8
c-Myb [T00137]; 8
NF-AT2 [T01945]; 10
TFII-I [T00824]; 6
STAT4 [T01577]; 6
c-Ets-1 [T00112]; 7
STAT1beta [T01573]; 10
IRF-1 [T00423]; 9
NF-AT1 [T00550]; 9
XBP-1 [T00902]; 6
Pax-5 [T00070]; 7
p53 [T00671]; 7
PEA3 [T00685]; 9
c-Jun [T00133]; 7
YY1 [T00915]; 4
PPAR-alpha:RXR-alpha [T05221]; 11
HNF-1C [T01951]; 9
HNF-1B [T01950]; 9
PR B [T00696]; 7
PR A [T01661]; 7
AP-1 [T00029]; 9
GATA-1 [T00306]; 6
IRF-2 [T01491]; 6
```
