# Supplementary material for: Midkine promotes renal fibrosis by stabilizing C/EBPβ to facilitate endothelial-mesenchymal transition
Source: Commun Biol. 2024 May 7;7:544. doi: 10.1038/s42003-024-06154-0 (PMC11076470; doi:10.1038/s42003-024-06154-0)
Supplement: Supplementary file 2 — Description of Additional Supplementary Files [file 42003_2024_6154_MOESM2_ESM.pdf]

Description of Additional Supplementary Files

File name: Supplementary Data 1

Description: All data source underlying the graphs and charts
