# Supplementary material for: Midkine promotes renal fibrosis by stabilizing C/EBPβ to facilitate endothelial-mesenchymal transition
Source: Commun Biol. 2024 May 7;7:544. doi: 10.1038/s42003-024-06154-0 (PMC11076470; doi:10.1038/s42003-024-06154-0)
Supplement: Supplementary file 4 — reporting-summary [file 42003_2024_6154_MOESM4_ESM.pdf]

nature portfolio

Corresponding author(s): Ruiming Rong

Last updated by author(s): Mar 3, 2024

Reporting Summary

Nature Portfolio wishes to improve the reproducibility of the work that we publish. This form provides structure for consistency and transparency in reporting. For further information on Nature Portfolio policies, see our Editorial Policies and the Editorial Policy Checklist.

Statistics

For all statistical analyses, confirm that the following items are present in the figure legend, table legend, main text, or Methods section.

n/a	Confirmed	
☐	☒	The exact sample size (*n*) for each experimental group/condition, given as a discrete number and unit of measurement
☐	☒	A statement on whether measurements were taken from distinct samples or whether the same sample was measured repeatedly
☐	☒	The statistical test(s) used AND whether they are one- or two-sided<br>*Only common tests should be described solely by name; describe more complex techniques in the Methods section.*
☒	☐	A description of all covariates tested
☐	☒	A description of any assumptions or corrections, such as tests of normality and adjustment for multiple comparisons
☐	☒	A full description of the statistical parameters including central tendency (e.g. means) or other basic estimates (e.g. regression coefficient) AND variation (e.g. standard deviation) or associated estimates of uncertainty (e.g. confidence intervals)
☐	☒	For null hypothesis testing, the test statistic (e.g. *F*, *t*, *r*) with confidence intervals, effect sizes, degrees of freedom and *P* value noted<br>*Give P values as exact values whenever suitable.*
☒	☐	For Bayesian analysis, information on the choice of priors and Markov chain Monte Carlo settings
☒	☐	For hierarchical and complex designs, identification of the appropriate level for tests and full reporting of outcomes
☒	☐	Estimates of effect sizes (e.g. Cohen's *d*, Pearson's *r*), indicating how they were calculated

Our web collection on statistics for biologists contains articles on many of the points above.

Software and code

Policy information about availability of computer code

Data collection	Sequence data were produced by Illumina NextSeq6000
Data analysis	The scRNA data were processes with Cell Ranger 3.0.1 and Seurat 4.1.1 in R (version 4.1.2) with less than 200 UMIs. The data was indenpently scaled and normalized and then merged to create a single-cell reference with Harmony. The monocle2 (v.2.4.0) algorithm was used to construct pseudotime trajectory. Cellahcat package was used to analyze the cell-cell communications.<br>The spatial transcriptomics were mapped using Space Ranger V1.1.0. and were processed by Seurat 4.1.1. The data were further normalzed by SCTransform and merged to build a unified UMAP.<br>Numerical computing and statistical analysis were conducted by Graphpad 9.0<br>Western blots were analyzed by Image J (V1.8.0)

For manuscripts utilizing custom algorithms or software that are central to the research but not yet described in published literature, software must be made available to editors and reviewers. We strongly encourage code deposition in a community repository (e.g. GitHub). See the Nature Portfolio guidelines for submitting code & software for further information.

Data

Policy information about availability of data

All manuscripts must include a data availability statement. This statement should provide the following information, where applicable:

- Accession codes, unique identifiers, or web links for publicly available datasets
- A description of any restrictions on data availability
- For clinical datasets or third party data, please ensure that the statement adheres to our policy

The spatial and scRNA datasets generated as a part of this study have been deposited in the Genome Sequence Archive58 in National Genomics Data Center59, China National Center for Bioinformation/Beijing Institute of Genomics, Chinese Academy of Sciences (GSA: HRA006794, HRA006796) that are publicly accessible at https://ngdc.cncb.ac.cn/gsa. The source data for the graphs in this study are provided in Supplementary Data. The WB uncropped images are provided in Supplementary Fig 5 in Supplementary Information. The predicted results of possible transcriptions factor of ACTA2 by online databate PROMO are provided in Supplementary Fig 6 in Supplementary Information. The authors declare that all other data supporting the findings of this study are available within the article and its supplementary information files, or are available from the authors upon request.

Research involving human participants, their data, or biological material

Policy information about studies with human participants or human data. See also policy information about sex, gender (identity/presentation), and sexual orientation and race, ethnicity and racism.

Reporting on sex and gender	N/A
Reporting on race, ethnicity, or other socially relevant groupings	N/A
Population characteristics	N/A
Recruitment	N/A
Ethics oversight	N/A

Note that full information on the approval of the study protocol must also be provided in the manuscript.

Field-specific reporting

Please select the one below that is the best fit for your research. If you are not sure, read the appropriate sections before making your selection.

☒ Life sciences ☐ Behavioural & social sciences ☐ Ecological, evolutionary & environmental sciences

For a reference copy of the document with all sections, see nature.com/documents/nr-reporting-summary-flat.pdf

Life sciences study design

All studies must disclose on these points even when the disclosure is negative.

Sample size	No statistical method was used to predetermine sample size. Sample sizes were chosen based on those used in previous and preliminary studies and preliminary studies from our lab which allow for statistical analysis. Sample sizes are indicated in the Figure, Figure legends or main text.
Data exclusions	No data were excluded from study
Replication	Numbers of replicates were indicated in the figure legends and method section.
Randomization	Male C57BL/6 mice, 6-8 weeks of age, were randomly assigned to sham operation or UUO model using a single sequence of simple randomization assignments. No randomization was done for the other experiments.
Blinding	Investigators were blinded to group allocation during data collection and analysis.

Reporting for specific materials, systems and methods

We require information from authors about some types of materials, experimental systems and methods used in many studies. Here, indicate whether each material, system or method listed is relevant to your study. If you are not sure if a list item applies to your research, read the appropriate section before selecting a response.

Materials & experimental systems

n/a	Involved in the study
☐	☒ Antibodies
☐	☒ Eukaryotic cell lines
☒	☐ Palaeontology and archaeology
☐	☒ Animals and other organisms
☐	☒ Clinical data
☒	☐ Dual use research of concern
☒	☐ Plants

Methods

n/a	Involved in the study
☒	☐ ChIP-seq
☒	☐ Flow cytometry
☒	☐ MRI-based neuroimaging

Antibodies

Antibodies used	VWF (Proteintech, Cat#11778-1-AP, WB: 1:1000)<br>ACTA2 (Proteintech, Cat#14395-1-AP, WB: 1:1000, IF: 1:100)<br>DCN (ABclonal, Cat#A1669, WB: 1:1000)<br>β-actin (Cell Signaling Technology, Cat#8457S, WB: 1:5000)<br>GAPDH (Cell Signaling Technology, Cat#2118S, WB: 1:1000)<br>C/EBPβ(Cell signaling Technology, Cat#43095S, WB: 1:1000, IF:1:100, IP: 1:50)<br>MDK (Proteintech Cat#11009-1-AP, WB: 1:1000, IF: 1：100)<br>Col1a1 (Cell Signaling Technology, Cat#72026, WB: 1:1000)<br>CD31 (Proteintech, Cat#10201-2-AP, WB: 1:1000)<br>His (Proteintech, Cat#66005-1-Ig, IP: 1:50)
Validation	All the commercially available antibodies were vadidated by the manufacturer via immunoblot or IF imaging.

Eukaryotic cell lines

Policy information about cell lines and Sex and Gender in Research

Cell line source(s)	HUVECs (purchased from Merck)
Authentication	The cell line used in this study was authenticated using STR profiling
Mycoplasma contamination	Comfirmed that all cells were negative for mycoplasma contamunation by manufacturer
Commonly misidentified lines<br>(See ICLAC register)	None

Animals and other research organisms

Policy information about studies involving animals; ARRIVE guidelines recommended for reporting animal research, and Sex and Gender in Research

Laboratory animals	Mdk-/- mice, Mdkflox/flox mice (Mdkfl/fl) and vascular endothelial-cadherin Cre recombinase-positive (CDH5-Cre) mice were purchased from Cyagen (Shanghai, China). Endothelial-specific Mdk knockout mice were generated by crossing CDH5-Cre mice and Mdkfl/fl mice with loxP sites flanking exon3 of Mdk gene. Male C57BL/6 mice were purchased from Cyagen (Shanghai, China). All animals were maintained under constant humidity and temperature at standard facilities under SPF conditions with free access to food and water.
Wild animals	The study did not involved in wild animals
Reporting on sex	The male animals were used in this study
Field-collected samples	No Field-collected samples were used in this study
Ethics oversight	All animal experiments were performed in accordance with protocols approved by the Animal Ethics Committee of Zhongshan Hospital, Fudan University.

Note that full information on the approval of the study protocol must also be provided in the manuscript.

Clinical data

Policy information about clinical studies

All manuscripts should comply with the ICMJE guidelines for publication of clinical research and a completed CONSORT checklist must be included with all submissions.

Clinical trial registration	B2020-046R, B2020-050R
Study protocol	All clinical procedures were approved by the Ethics Committee of Zhongshan Hospital, Fudan University
Data collection	In scRNA-seq, three fibrosis tissues were obtained from a renal transplant patient who had biopsy-proven interstitial fibrosis. The fibrotic tissues were obtained from a patient's nonfunction kidney secondary to high-grade obstruction. The control samples were obtained from patients who received nephrectomy because of renal cancer without diabetes or chronic kidney disease.<br>In spatial transcriptomics, we collected tissue from a 53-years-old woman with interstitial fibrosis for spatial transcriptomics.
Outcomes	The fibrosis tissues were obtained from patient who had biopsy-proven interstitial fibrosis. The non-fibrosis samples were obtained from patients who received nephrectomy because of renal cancer without CKD.

Plants

Seed stocks	*Report on the source of all seed stocks or other plant material used. If applicable, state the seed stock centre and catalogue number. If plant specimens were collected from the field, describe the collection location, date and sampling procedures.*
Novel plant genotypes	*Describe the methods by which all novel plant genotypes were produced. This includes those generated by transgenic approaches, gene editing, chemical/radiation-based mutagenesis and hybridization. For transgenic lines, describe the transformation method, the number of independent lines analyzed and the generation upon which experiments were performed. For gene-edited lines, describe the editor used, the endogenous sequence targeted for editing, the targeting guide RNA sequence (if applicable) and how the editor was applied.*
Authentication	*Describe any authentication procedures for each seed stock used or novel genotype generated. Describe any experiments used to assess the effect of a mutation and, where applicable, how potential secondary effects (e.g. second site T-DNA insertions, mosiacism, off-target gene editing) were examined.*
